# External Mechanical Stability Regulates Hematoma Vascularization in Bone Healing Rather than Endothelial YAP/TAZ Mechanotransduction

**DOI:** 10.1002/advs.202307050

**Published:** 2024-01-25

**Authors:** Julia Mehl, Saeed Khomeijani Farahani, Erik Brauer, Alexandra Klaus‐Bergmann, Tobias Thiele, Agnes Ellinghaus, Eireen Bartels‐Klein, Katharina Koch, Katharina Schmidt‐Bleek, Ansgar Petersen, Holger Gerhardt, Viola Vogel, Georg N. Duda

**Affiliations:** ^1^ Julius Wolff Institute Berlin Institute of Health at Charité – Universitätsmedizin Berlin 13353 Berlin Germany; ^2^ Berlin Institute of Health Center for Regenerative Therapies Berlin Institute of Health at Charité – Universitätsmedizin Berlin 13353 Berlin Germany; ^3^ Laboratory of Applied Mechanobiology Department of Health Sciences and Technology ETH Zurich Zurich 8092 Switzerland; ^4^ Integrative Vascular Biology Laboratory Max‐Delbrück‐Center for Molecular Medicine (MDC) in the Helmholtz Association 13125 Berlin Germany; ^5^ German Center for Cardiovascular Research (DZHK) Partnersite Berlin 10785 Berlin Germany

**Keywords:** angiogenesis, bone healing, endothelial yes‐associated protein 1/transcriptional coactivator with PDZ‐binding motif, mechanobiology, mechanotransduction

## Abstract

Bone fracture healing is regulated by mechanobiological cues. Both, extracellular matrix (ECM) deposition and microvascular assembly determine the dynamics of the regenerative processes. Mechanical instability as by inter‐fragmentary shear or compression is known to influence early ECM formation and wound healing. However, it remains unclear how these external cues shape subsequent ECM and microvascular network assembly. As transcriptional coactivators, the mechanotransducers yes‐associated protein 1 (YAP)/transcriptional coactivator with PDZ‐binding motif (TAZ) translate physical cues into downstream signaling events, yet their role in sprouting angiogenesis into the hematoma after injury is unknown. Using bone healing as model system for scar‐free regeneration, the role of endothelial YAP/TAZ in combination with tuning the extrinsic mechanical stability via fracture fixation is investigated. Extrinsically imposed shear across the gap delayed hematoma remodeling and shaped the morphology of early collagen fiber orientations and microvascular networks, suggesting that enhanced shear increased the nutrient exchange in the hematoma. In contrast, endothelial YAP/TAZ deletion has little impact on the overall vascularization of the fracture gap, yet slightly increases the collagen fiber deposition under semi‐rigid fixation. Together, these data provide novel insights into the respective roles of endothelial YAP/TAZ and extrinsic mechanical cues in orchestrating the process of bone regeneration.

## Graphical abstract

Novel insights into the mechano‐regulation of early bone healing by fixation stability or endothelial yes‐associated protein 1 (YAP)/transcriptional coactivator with PDZ‐binding motif (TAZ) are presented. Mechanical (in‐)stability of the osteotomy gap significantly shapes vascular and collagen network morphologies during hematoma remodeling. Contrary, endothelial YAP/TAZ has a minor impact on vascularization, but regulates extracellular matrix formation potentially via paracrine signaling.

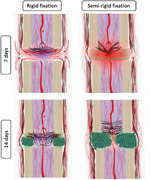

## 1 Introduction

Bone is one of the few organs that has the capability to heal scar‐free. To reach scar‐free healing after fracture, however, a certain amount of fragment stabilization is required with little compression of fracture fragments and minimal or no shear across the fracture gap.^[^ ^1^, ^2^ ^]^ Lack or insufficient fracture fixation prevents proper bone formation or leads to delayed healing which can cause scarring.^[^ ^1^, ^3^ ^]^ While bone remodeling locally is known to adapt to extrinsically imposed mechanical stress since Wolff's initial observations,^[^ ^4^ ^]^ how cellular mechanotransduction processes regulate early bone healing remains elusive.

Upon bone fracture or surgical osteotomy, nutrition supply is abruptly interrupted for all cells residing in the fracture or osteotomy zone thus creating a hypoxic environment.^[^ ^5^ ^]^ Recent studies revealed sequential events and cellular mechanisms that orchestrate the healing process.^[^ ^6^, ^7^, ^8^ ^]^ Once platelets are exposed to damaged vasculature, they become activated and form a fibrin clot filling the osteotomy gap, ultimately stopping the bleeding.^[^ ^9^ ^]^ As the fibrin clot forms, platelets together with other blood‐borne cells including red blood cells and immune cells become entrapped.^[^ ^10^ ^]^ These are the first to start secreting growth factors and cytokines during the acute pro‐inflammatory phase, further recruiting other inflammatory cells, mesenchymal stromal cells (MSCs), fibroblasts, as well as endothelial cells (EC) from the surrounding soft tissue, bone marrow, cortex, and periosteum.^[^ ^11^, ^12^, ^13^ ^]^ Concomitantly, staged remodeling of the hematoma is initiated,^[^ ^14^ ^]^ whereby platelets contract the fibrin clot and assemble the very first provisional fibronectin fibers within the hematoma.^[^ ^15^ ^]^ The blood clot with entrapped blood cells in synergistic interactions with invading fibroblasts and MSCs secrete factors associated with fast healing such as the pro‐angiogenic vascular endothelial growth factor (VEGF) and matrix metalloproteinases (MMPs).^[^ ^10^ ^]^ While ECs invade the hematoma upon VEGF secretion and form new capillaries, invading stromal cells, including fibroblasts and MSCs, deposit extracellular matrix (ECM). These events lead to the formation of granulation tissue, gradually degrading the hematoma and replacing it with new tissue.^[^ ^16^ ^]^ Moreover, MSCs have an immunosuppressive character, which helps to resolve the initial acute inflammation.^[^ ^17^ ^]^ MSCs within the granulation tissue have the potential to differentiate into osteoblasts or chondrocytes. Relevant factors for fate decisions are mechanical factors and oxygen tension.^[^ ^18^ ^]^ Mechanical stress is known to induce chondrocyte differentiation within the fracture gap by condensation of progenitors.^[^ ^19^ ^]^ This paves the way for the stabilization of fractures and the replacement of granulation tissue by endochondral ossification.^[^ ^20^ ^]^ Also proliferating and differentiating chondrocytes release VEGF to attract blood vessels to invade the forming cartilage islands.^[^ ^21^ ^]^ Later, endothelial sprouts penetrate avascular cartilage islands and promote osteoprogenitor recruitment to allow a later re‐organization of a cartilaginous callus.^[^ ^22^ ^]^ Finally, a mineralized callus is quickly formed from woven bone, which later is remodeled to lamellar bone with a highly ordered collagen matrix.^[^ ^16^ ^]^

After injury, the re‐establishment of the vasculature is of utmost importance to enable tissue reconstitution.^[^ ^23^ ^]^ Re‐establishing a microvascular network as the hematoma gets remodeled, ultimately bridges the injury site and is essential for later maturation and remodeling stages. While this general relevance of angiogenesis in tissue remodeling is widely accepted, how early ECM architecture and preferential fiber alignment are formed and how this is linked to the spatiotemporal initiation of angiogenesis, vessel structuring, and re‐organization remains poorly understood.

Wound repair is accompanied by a massive influx of fibroblasts, other mesenchymal progenitors, and immune cells from the surrounding tissues into the hematoma‐filled site of injury.^[^ ^16^ ^]^ Various cell types become mobilized and actively migrate into the site of injury, typically guided by topographical cues provided by ECM fibers. In addition to extrinsically acting forces, platelets, and entrapped and invading cells all generate traction forces by which they pull on neighboring cells and on the surrounding ECM. They thereby sense and respond also to physical properties of their environment such as viscoelasticity and substrate stiffness, but also topography and the presence of fibers.^[^ ^24^, ^25^, ^26^ ^]^ This eventually leads to cytoskeletal assembly and re‐arrangements, which in turn stabilizes the cell‐ECM adhesions via integrins.^[^ ^27^, ^28^ ^]^ One key mechanism for cells to alter their gene transcription in response to mechanical cues is through the nuclear transcriptional coactivators of the Hippo signaling pathway, that is, the yes‐associated protein 1 (YAP) and transcriptional coactivator with PDZ‐binding motif (TAZ). YAP/TAZ senses the mechanical properties of a cell's environment and translocates to the nucleus when the cytoskeleton gets tensed, where it controls genes responsible for cell proliferation, apoptosis, cell motility, and cell fate decisions through interaction with, that is, TEA domain family member (TEAD) transcription factors.^[^ ^29^, ^30^, ^31^ ^]^ YAP/TAZ thus play a central role as mechanotransducer for cell‐cell and cell‐matrix interactions, and as an important regulator of microvascular network patterning and permeability.^[^ ^32^ ^]^ In the context of bone healing, deletion of YAP/TAZ in pre‐osteoblasts after normal development, but before fracture, has been shown to impair the proliferation of pre‐osteoblasts in the early stages of bone healing and reduce bone formation 14 days after fracture.^[^ ^33^ ^]^ Further, it has been shown that endothelial YAP/TAZ suppresses vascular growth in bone growth plates by inhibiting HIF‐1α gene expression.^[^ ^34^ ^]^ However, insights into the relevance of YAP/TAZ in endothelial sprouting angiogenesis during in vivo regeneration in a mechanically demanding setting such as an osteotomy gap remain elusive. We thus asked how endothelial YAP/TAZ might affect bone healing. To answer this question, we specifically deleted YAP/TAZ in ECs of mice undergoing bone healing while they freely moved in the cage. On a macroscopical level, the quality of bone healing is influenced by the local fracture fixation stability, whereby too little stability can delay or impair bone healing.^[^ ^1^, ^3^ ^]^ A mechanically induced delay in bone healing has been shown to prolong inflammation and all successive stages of healing from relatively early stages and onwards.^[^ ^3^ ^]^

Here, we investigated how externally imposed interfragmentary movement via a semi‐rigid fixator versus a rigid fixator and endothelial YAP/TAZ affect collagen fiber assembly, microvessel orientations, and EC adherens junctions, and thereby the overall bone healing processes. We initially hypothesized that microvascular sprouting in early hematoma remodeling might be modulated by endothelial YAP/TAZ.

## 2 Results

**Figure 1:**
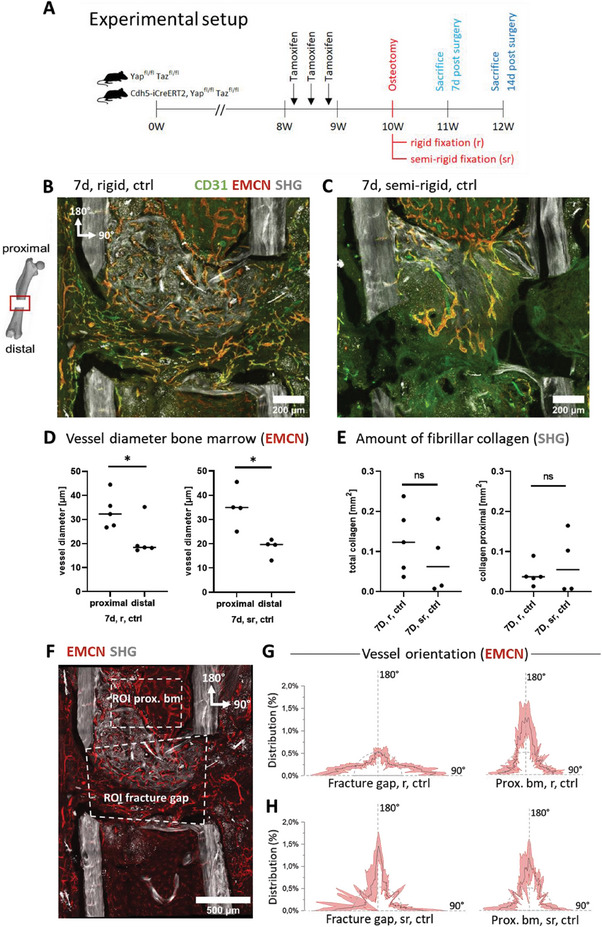
Delayed hematoma remodeling under semi‐rigid fixation, with only a few vessels penetrating the gap and with different vessel orientations compared to rigid fixation. A) Schematic showing the experimental setup. Control mice (Yap^fl/fl^ Taz^fl/fl^), as well as endothelial YAP/TAZ dKO mice (Cdh5‐iCreERT2, Yap^fl/fl^ Taz^fl/fl^), received tamoxifen before osteotomy was placed in the left femur of the mice. Osteotomy was set in 10‐weeks‐old female mice and stabilized with either a rigid or semi‐rigid fixator. Mice were sacrificed 7 or 14 days post‐osteotomy. SHG imaging was performed to visualize cortical bone and collagen fibers in the osteotomy gap. Immunofluorescence staining was performed for EMCN and CD31 to visualize the vascular system including blood clot entrapped platelets. Maximum projection images were acquired by confocal microscopy. B) Representative image of fully vascularized hematoma with rigid fixation. Vessels positive for EMCN and CD31 could be identified as venous vessels, whereas only CD31 positive vessels as arteries. Vessel ingrowth mainly appeared from the proximal bone marrow side and adjacent soft tissue. SHG imaging revealed fibrillar collagen deposition on the proximal side of the gap, building a bridge spanning the two proximal cortical fracture ends. Collagen fibers were mainly oriented perpendicular to the cortical bone axis (scale bars = 200 µm). C) Mechanically challenged osteotomy gap of mice by fixation with a semi‐rigid fixator. The representative image revealed that the osteotomy gap was still mainly filled with the hematoma illustrated by the greenish glowing background, specifically on the distal side of the gap, emerging from platelets that stained positive for CD31. Only a few vessels penetrated the gap from the proximal side. SHG images revealed fibrillar collagen deposition mainly within the proximal bone marrow cavity between the proximal cortical fracture ends. The increased interfragmentary movement in semi‐rigid fixation led to collagen fibers oriented more parallel to the cortical bone axis compared to rigid (scale bar = 200 µm). D) Quantification of mean vessel diameter of EMCN‐positive vessels in the proximal bone marrow compared to distal bone marrow. Microvascular networks staining for EMCN were thicker in diameter on the proximal bone marrow side compared to the one within the distal bone marrow with both, rigid and semi‐rigid fixator, indicating a proximal to distal polarization in bone healing (*N* = 5 for rigid, *N* = 4 for semi‐rigid). E) Fibrillar collagen area by SHG in the fracture gap and proximal bone marrow cavity (total collagen) and proximal bone marrow cavity only (proximal collagen). Statistical analysis using unpaired, parametric *t*‐tests was performed. *p*‐value was indicated by * for *p* < 0.05. F) Exemplary image (rigid fixation) showing different regions of interest as used in our analyses (ROI proximal bone marrow, ROI fracture gap). 180° is defined as parallel to the central bone axis and 90° degree perpendicular to the central bone axis (scale bar = 500 µm). G,H) Orientation analysis of vessels in the osteotomy gap for G) rigid and H) semi‐rigid fixations and proximal bone marrow. G) Orientation plots show that under rigid fixation vessels were mostly aligned perpendicular to the bone axis in the osteotomy gap. In contrast, vessels that grew in from the bone marrow mostly aligned parallel to the bone axis (*N* = 5). H) Under semi‐rigid vessels shifted their orientation and aligned more parallel to the bone axis. Vessels in the bone marrow remained parallel to the bone axis (*N* = 4).

We selected 7 days post‐osteotomy as an early time point, which presents the end of the inflammatory phase of bone healing.^[^ ^6^, ^35^ ^]^ At this stage, the space occupied by thrombus‐entrapped platelets and blood‐borne cells showed early collagen fiber network organization and the re‐establishment of first vascular structures. The healing processes in these early 0.7 mm wide osteotomy gaps were analyzed here between the proximal and distal cortical ends. The blood‐borne cells entrapped in the hematoma were visualized via platelet adhesion molecule‐1 (PECAM‐1), which is expressed on the surface of circulating platelets and monocytes,^[^ ^36^, ^37^ ^]^ also commonly known as CD31. As CD31 is also present on the surface of ECs, and since the number of markers is limited that we could combine in each stain, CD31 was also used to monitor the progression of venous and arterial microvascular networks together with the EC marker endomucin (EMCN), which only stains venous capillaries.^[^ ^38^ ^]^ The superimposition of CD31 and EMCN‐positive vessels are known as H‐type vessels and are important mediators in the coupling of osteogenesis and angiogenesis.^[^ ^39^ ^]^ Thick bundles of fibrillar collagen were visualized by second harmonic generation (SHG) imaging.^[^ ^40^ ^]^ Next to analyzing collagen matrix assembly and vascular development, we identified early signs of bone formation by staining for the transcription factor osterix (OSX), a known key mediator of osteoblast differentiation and osteogenesis‐angiogenesis coupling.^[^ ^39^ ^]^ **Figure** **1**A illustrates the experimental setup used in our study.

### 2.1 Blood Vessels Fully Vascularized the Hematoma‐Filled Gap in the Early Stage of Bone Healing upon Rigid Fixation

Since vascularization of the hematoma is an early event and a pre‐requirement for successful bone healing, we asked how the vascular network developed in the hematoma‐filled gap. We used a fixation known to show scar‐free healing at 14 days with complete bone bridging at 21 days (“rigid fixation”).^[^ ^41^, ^42^ ^]^ Figure 1B shows one representative sample of the 7‐day time point under such rigid fixation conditions illustrating that a microvascular network of CD31 and EMCN‐positive vessels already spanned the complete osteotomy gap, mostly perpendicular to the bone axis. To analyze collagen matrix assembly and organization, we further performed SHG imaging to visualize thick fibrillar collagen bundles. In contrast to the vascular network spanning the whole gap, fibrillar collagen was mostly present on the proximal side of the gap. In contrast, the blood vessels at the distal side were still surrounded by a faint CD31‐rich background, suggesting that they were still surrounded by a platelet‐rich hematoma. Our images revealed different sources of vessel ingrowth, which mainly emerged from the proximal bone marrow, or alternatively from the soft tissues that surrounded the bone and fracture gap. Noteworthy, many ingrowing vessels coming from the surrounding soft tissues were of arterial nature, staining only positive for CD31, and penetrated the partially remodeled hematoma at the distal side of the gap (Figure 1B).

### 2.2 Rigid Fixation Permitted Gap Bridging by Collagen Fibers on the Proximal Side

At 7 days post‐osteotomy, SHG imaging revealed that fibrillar collagen bundles were only partially present in the osteotomy gap with considerable variations from mouse to mouse (Figure S1A, Supporting Information). SHG positive collagen fibers were found mostly assembled at the proximal side. In three out of five samples, collagen fibers bridged the cortical fracture ends (Figure S1A, Supporting Information). Thus, the newly formed collagen fibers spanned the bone cortices preferentially perpendicular to the highly organized cortical collagen matrix of the lamellar bone. In contrast, no such bridging was seen at the distal cortical ends. In none of the samples did SHG‐positive collagen fibers bridge the whole fracture gap from the proximal to the distal side (Figure S1A, Supporting Information).

### 2.3 Increased Shear in Semi‐Rigid Fixation Delayed Hematoma Remodeling with Only Few Vessels Penetrating the Gap

**Figure 2:**
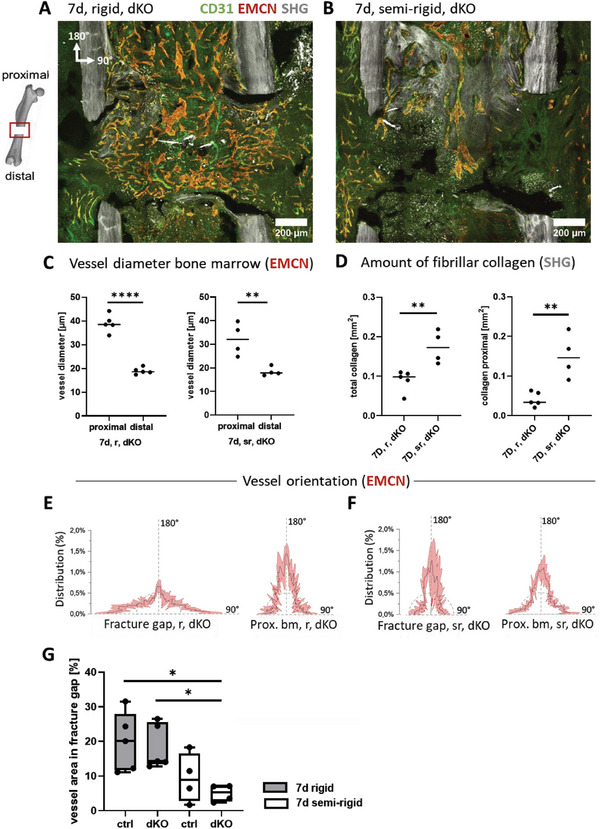
Endothelial YAP/TAZ knockout had little effect on vascularization under rigid as well as semi‐rigid fixation. Conditional knockout of YAP/TAZ in ECs was performed to ask how compromised mechanosensation of ECs might impact the vascularization of the fracture gap in the early phases of bone healing. A) As for control mice, the gap was fully vascularized with venous and some arterial vessels, indicating a fully vascularized hematoma. SHG imaging revealed fibrillar collagen deposition on the proximal side of the gap, starting to build a bridge spanning the two proximal cortical fracture ends (scale bars = 200 µm). B) Representative image for semi‐rigid fixation showing delayed hematoma remodeling even when endothelial‐specific YAP/TAZ was deleted. SHG imaging revealed fibrillar collagen deposition mainly within the proximal bone marrow cavity between the proximal cortical fracture ends, as seen also for the control mice (Figure 1C) (scale bars = 200 µm). Thus, external mechanics mostly steer vascular network morphology with little impact on endothelial YAP/TAZ mechanosensing. Vessels in the bone marrow remained parallel to the bone axis (*N* = 4). C) Quantification of mean vessel diameter at proximal bone marrow compared to distal bone marrow. Microvascular networks staining for EMCN were thicker in diameter on the proximal bone marrow side compared to the one within the distal bone marrow with both, rigid and semi‐rigid fixators. This indicates a proximal to distal polarization in bone healing also upon endothelial YAP/TAZ deletion. D) Fibrillar collagen area by SHG in the fracture gap and proximal bone marrow cavity (total collagen) and proximal bone marrow cavity only (proximal collagen). Total collagen area and collagen area within the proximal bone marrow cavity significantly increased under semi‐rigid fixation compared to rigid. Statistical analysis using unpaired, parametric *t*‐tests was performed. *p*‐value was indicated by ** for *p* < 0.01 and **** for *p* < 0.0001. E,F) Orientation analysis of vessels in the osteotomy gap for E) rigid and F) semi‐rigid fixations and proximal bone marrow. 90° indicates perpendicular to the central bone axis, 180° parallel to the central bone axis. E) Orientation plots show that vessels were mostly aligned perpendicular to the bone axis in the osteotomy gap under rigid fixation. In contrast, vessels that grew in from the bone marrow mostly aligned parallel to the bone axis (*N* = 5). F) Under semi‐rigid vessels shifted their orientation and aligned more parallel to the bone axis like control (Figure 1H). G) Comparison of vessel area within the fracture gap for control and knockout mice at the 7‐day time‐point. Statistical analysis using one‐way ANOVA followed by Tukey's test was performed. *p*‐value was indicated by * for *p* < 0.05 (*N* = 4–5).

To verify whether the speed of hematoma remodeling depends on the mechanical stability of the fracture fixation, we chose a bone healing model with increased interfragmentary movement due to a less stable fixation (“semi‐rigid fixation”).^[^ ^3^ ^]^ Compared to rigid fixation, the mechanically challenged fracture gap showed delayed hematoma remodeling under semi‐rigid fixation as illustrated by a lack of blood vessels, while a diffuse signal of CD31‐positive platelets was still visible at the distal side of the gap (Figure 1C). While microvascular networks had formed at the proximal bone marrow cavity, they only partially penetrated the osteotomy gap on the distal side. In contrast to rigid fixation, vessels penetrating the osteotomy gap mostly originated from the proximal bone marrow rather than from the outside (Figure 1C). The observation of reduced and eventually delayed formation of a microvasculature confirmed findings from earlier work in large animals.^[^ ^43^, ^44^ ^]^ This was further quantified by measuring the vessel density within the osteotomy gap upon rigid versus semi‐rigid fixations (**Figure** **2**G). A proximal to distal polarization of the microvasculature was detected under both, rigid and semi‐rigid fixations and quantified by measuring the mean vessel diameter at the proximal compared to the distal bone marrow side (Figure 1D).

We further asked whether the formation of a collagen matrix is perturbed at 7 days post‐osteotomy under semi‐rigid fixation. As in rigid fixation, SHG‐positive collagen fiber deposition was prominent on the proximal side of the gap, also under semi‐rigid fixation (Figure S1E, Supporting Information). However, strikingly, the collagen fiber bundles were running more parallel to the long bone axis rather than bridging the proximal bones as observed under rigid fixation. Figure 1E shows the quantification of total SHG‐positive collagen and SHG‐positive collagen on the proximal bone marrow side illustrating the heterogeneity across mice at this early stage of bone healing under rigid and semi‐rigid fixation.

### 2.4 Increased Mechanical Shear Across the Gap Caused Different Vessel Orientations Compared to Rigid Fixation

To further investigate the different morphologies of the vascular networks under different external mechanical stabilities, we quantified vessel orientations in distinct ROIs (Figure 1F), again highlighting the remarkable shift in their orientations towards the long bone axis under semi‐rigid compared to rigid fixation (Figure 1G,H). Under semi‐rigid fixation though, the microvascular patterns appeared to be more heterogeneous across mice compared to rigid (Figure 1G,H). In both, rigid and semi‐rigid fixations, vessels penetrating the gap from the proximal bone marrow aligned parallel to the central bone axis (Figure 1G,H). For the first time, we hereby show that the stability of the external fixation not only controls hematoma remodeling but also significantly shapes the morphology of the emerging vascular network.

### 2.5 Overall Vascularization of the Hematoma was not Affected by Endothelial YAP/TAZ Deletion

ECs require anchorage to ECM fibers for migration and vascularization of the hematoma. Vascular sprouting is facilitated by EC migration along fibers, as mediated by fiber adhesion via integrins, cytoskeletal arrangements, and actin filament remodeling, as well as firm EC‐EC contacts.^[^ ^45^, ^46^, ^47^ ^]^ Since nuclear translocation of YAP/TAZ is also triggered by enhanced cell contractility,^[^ ^31^ ^]^ we next asked how depletion of endothelial YAP/TAZ would affect the growth of microvascular structures and hence hematoma remodeling. We therefore specifically depleted endothelial YAP/TAZ by using the well‐established *Yap^fl/fl^ Taz^fl/fl^ Cdh5‐iCreERT2* mouse strain.^[^ ^48^, ^49^ ^]^ Cre‐mediated loss‐of‐function was induced by tamoxifen injection prior to osteotomy (Figure 1A). For recombination efficiency, genomic DNA from bone marrow and growth plate were used and genotyping PCRs were performed (Figure S2, Supporting Information). With endothelial‐specific YAP/TAZ depletion, hereafter called “EC YAP/TAZ dKO”, the hematoma was unexpectedly still fully vascularized after 7 days upon rigid fixation, whereby the microvascular networks filled and spanned the whole gap in all samples analyzed (Figure 2A). Blood vessels were positioned along fibrillar collagen at the proximal side and most vessels in the gap came either from the proximal bone marrow or from the surrounding soft tissue. Also under semi‐rigid fixation, EC YAP/TAZ dKO mice showed a similarly delayed hematoma remodeling compared to the control mice (Figure 2B). Even the proximal to distal polarization was little affected by EC YAP/TAZ dKO (Figure 2C). Upon YAP/TAZ deletion, the vessels again aligned preferentially perpendicular to the central bone axis under rigid fixation (Figure 2E), and parallel to the bone axis under semi‐rigid fixation (Figure 2F). This suggests that endothelial YAP/TAZ plays a minor role in enabling the vascularization of a hematoma in a bone fracture gap. The mechanical (in‐)stability of the osteotomy itself thus exerts a far more profound effect on microvascular network formation regardless of endothelial YAP/TAZ activity (Figure 2G).

Also, the morphology of the collagen fiber networks was similar for control and dKO mice, where SHG showed some collagen fibers surrounding the proximal cortices under rigid fixation, with essentially no fibrillar collagen at the distal side (Figure S1B, Supporting Information). Collagen fibers bridged the proximal cortices, however, only in one out of five samples (Figure S1B, Supporting Information). Interestingly though, quantification of the amount of fibrillar collagen revealed more collagen deposition under semi‐rigid compared to rigid fixation upon endothelial YAP/TAZ deletion (Figure 2D), which was not seen in the control mice (Figure 1E). This excess collagen was mostly found within the proximal bone marrow cavity under semi‐rigid fixation for the dKO mice.

### 2.6 OSX‐Expressing Cells Mainly Found Within the Collagen‐Rich Proximal Side of the Osteotomy Gap

**Figure 3:**
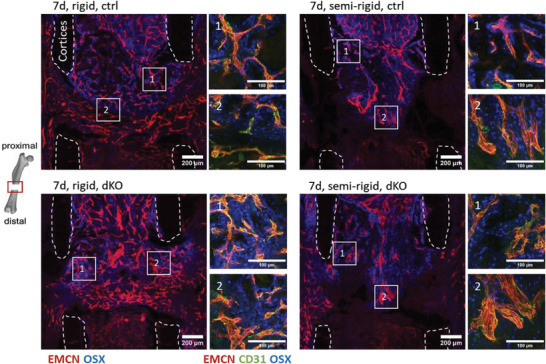
OSX expressing pre‐osteoblasts in proximity to vessels in early bone healing. Representative images of osteotomy gaps for OSX staining merged with EMCN staining. OSX expressing pre‐osteoblasts were found in proximity to vessels. They were upregulated in the SHG collagen‐rich environment in the proximal half of the gap, whereas fewer of them were found on the distal side, where still no collagen fibers by SHG were visible. Zoom‐in images show additional CD31 staining (scale bar = 200 µm; zoom‐in images: 100 µm).

OSX‐expressing pre‐osteoblasts are known to be in close proximity to vessels during bone formation in the growth plate,^[^ ^39^ ^]^ as they were reported to migrate along the outside wall of blood vessels,^[^ ^50^ ^]^ and are known to express collagen.^[^ ^51^ ^]^ Thus, we next stained for OSX within the hematoma‐filled gap. OSX‐expressing pre‐osteoblasts were mainly found in proximity to blood vessels (**Figure** **3**) and less in the fibrin‐rich distal side of the gap, suggesting that they play a role in osteogenesis‐angiogenesis coupling during the early phases of bone healing, as suggested before.^[^ ^52^ ^]^ OSX‐expressing cells were in proximity to vessels, even when depleting YAP/TAZ in ECs (Figure 3), illustrating their importance in mediating angiogenesis and osteogenesis in early bone healing.

### 2.7 EC YAP/TAZ Deletion Reduced VE‐Cadherin in Adherens Junctions in the Osteotomy Gap Compared to the Control

ECs pull via integrins on ECM substrates during sprouting angiogenesis which upregulates the tension of VE‐cadherin adhesive bonds,^[^ ^53^, ^54^, ^55^, ^56^, ^57^ ^]^ thereby regulating vascular development.^[^ ^58^ ^]^ Hence, we probed how EC YAP/TAZ deletion might impact VE‐cadherin EC junctions within the osteotomy gap. Under rigid fixation, representative images at 7 days post‐osteotomy showed significantly more faint VE‐cadherin junctions in EC YAP/TAZ dKO mice compared to the control (Figure S3, Supporting Information), suggesting that far less VE‐cadherin was recruited to the EC membrane in the absence of YAP/TAZ. Apparently, the EC junctions in newly forming vessels in the osteotomy gap are sensitive to the loss of endothelial YAP/TAZ similar to what has been reported in developmental angiogenesis.^[^ ^58^ ^]^ Since CD31 was found in EC‐EC contacts and has been suggested to act as a mechanosensor together with VE‐cadherin to maintain EC junctional integrity,^[^ ^59^, ^60^, ^61^ ^]^ we stained CD31 in addition to VE‐cadherin. Concomitant with VE‐cadherin, CD31 was also fainter at the EC‐EC junctions upon endothelial YAP/TAZ knockout compared to the control (Figure S3, Supporting Information).

### 2.8 Formation of Endochondral Ossification Islands Initiated a Second Phase of Angiogenesis

**Figure 4:**
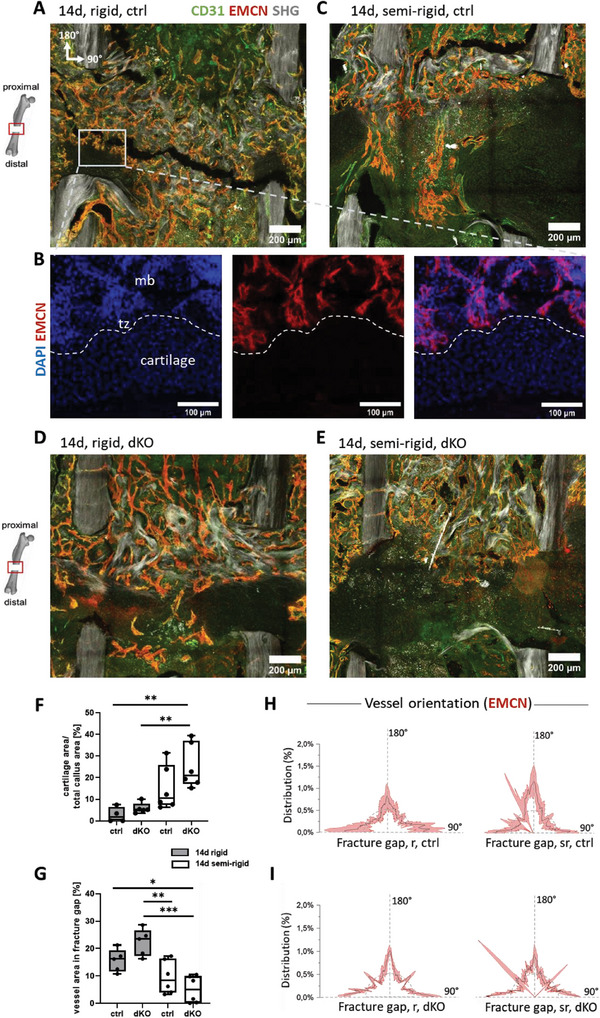
Cartilage development increased under semi‐rigid fixation compared to rigid, but was mostly independent of endothelial YAP/TAZ. Mice stabilized with a rigid or semi‐rigid fixator were sacrificed 14 days post‐osteotomy, which is known to be at the onset of endochondral ossification. A) Hematoma was replaced by vascularized tissue and cartilage tissue under rigid fixation as shown in the representative image of the osteotomy gap. Compared to 7 days, almost no arterial vessels could be identified (only CD31 positive vessels), but mostly venous vessels characterized by overlay of CD31 and EMCN staining. Collagen bridges that had formed at 7 days between cortices now reached further into the osteotomy gap. Avascular regions formed on the distal side of the gap flanking the cortical fracture ends, indicating the development of cartilage at these regions (scale bars = 200 µm). B) Zoom‐in image of bone‐cartilage transition zone with additional DAPI staining confirmed the presence of endochondral ossification in this region (mb = mineralized bone, tz = transition zone) (scale bars = 100 µm). C) Representative image for semi‐rigid fixation illustrating increased endochondral tissue formations with pronounced cartilage islands. Vessels started to bridge proximal to distal by passing through a not yet completely remodeled hematoma centrally to the bone axis. Collagen fiber deposition was primarily seen on the proximal bone marrow cavity only partially reaching into the gap (scale bars = 200 µm). D) Cartilage formation replaced microvascular networks on the distal end of the osteotomy gap even upon endothelial‐specific YAP/TAZ deletion under rigid fixation. E) Semi‐rigid fixation increased cartilage formation even upon endothelial YAP/TAZ deletion. F) Quantification of cartilage area over total callus area (*N* = 4–6). G) Comparison of vessel area within the fracture gap (*N* = 5–6). Statistical analysis using one‐way ANOVA followed by Tukey's test was performed. Different *p*‐values were indicated by *, **, and *** for *p* < 0.05, *p* < 0.01, and *p* < 0.001, respectively. H,I) Orientation analysis of vessels in the osteotomy gap for rigid fixation and semi‐rigid fixation. 180° indicates parallel to the central bone axis and 90° indicates perpendicular to the central bone axis. Orientation plots revealed that vessels experienced a broader distribution in their orientation under semi‐rigid compared to rigid fixation, in both, control and upon endothelial YAP/TAZ knockout. Thus, external mechanical stability steered vessel orientation within the osteotomy gap independent of endothelial YAP/TAZ (*N* = 5 for rigid, ctrl; rigid, dKO and semi‐rigid, ctrl; *N* = 3 for semi‐rigid, dKO).

Under rigid fixation, most bone fractures heal via endochondral ossification by ultimately forming a proximal to distal bone bridging.^[^ ^16^ ^]^ Under rigid fixation, the hematoma was replaced at 14 days by vascularized tissue rich in collagen in the osteotomy gaps, or by cartilage capping the distal ends of the bones (**Figure** **4**A). Movat's pentachrome staining confirmed the presence of cartilage tissue and the absence of vasculature in those distal regions (Figure S4, Supporting Information). With additional DAPI staining, a bone‐cartilage transition zone could be confirmed, verifying endochondral ossification (Figure 4B). Microvascular networks were again positioned adjacent to thick collagen fibers (Figure 4A). Under semi‐rigid fixation, blood vessels started to bridge from the proximal to the distal side in some samples by passing through a not‐yet‐completely remodeled hematoma (Figure 4C). In the remaining samples, the microvascular network only partially reached the osteotomy gap. An increased amount of cartilage was found within the healing region compared to the rigid condition. Movat's pentachrome staining confirmed the presence of cartilage islands, showing that cartilage had formed beneath the collagen and vascular front that came from the proximal side (Figure S5A, Supporting Information).

**Figure 5:**
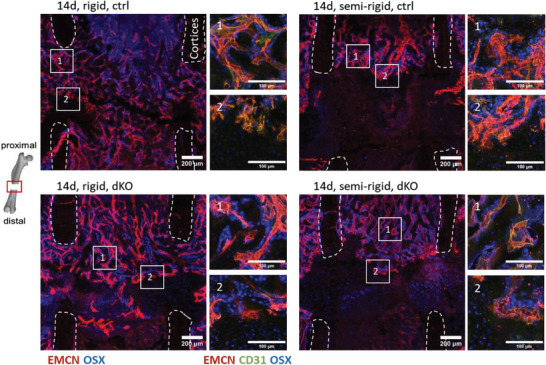
OSX‐expressing cells were in proximity to vessels, but only a few were in cartilage tissue at later stages of bone healing. Representative images of the osteotomy gaps of OSX staining superimposed with EMCN staining. OSX‐expressing cells were in proximity to vessels in the SHG collagen‐rich environment, whereas only a few OSX‐expressing cells could be found in cartilage areas. Zoom‐in images show additional staining of CD31. Image number 1 shows a zoom‐in of the collagen‐rich and vascularized tissue whereas image number 2 shows a zoom‐in image of the bone‐cartilage transition zone (scale bar = 200 µm; zoom‐in images: 100 µm).

Again, when comparing control with EC YAP/TAZ dKO mice after 14 days, microvascular networks formed adjacent to collagen fibers under rigid fixation (Figure 4D). Comparable or even more pronounced as in the control, cartilage developed mostly at the distal side of the gap where vessels were mostly absent (Figure 4D). This indicates that the main remodeling phases of bone repair were functional even under endothelial YAP/TAZ deletion. Semi‐rigid fixation in EC YAP/TAZ dKO mice led to increased avascular areas (Figure 4E), identified as cartilage islands as confirmed by Movat's pentachrome staining (Figure S5B, Supporting Information). Cartilage quantification revealed no significant difference in controls compared to endothelial YAP/TAZ knockouts under both, rigid and semi‐rigid conditions. However, the amount of cartilage significantly increased under semi‐rigid compared to rigid upon endothelial YAP/TAZ deletion (Figure 4F). Quantification of vessel areas in the fracture gap upon endothelial YAP/TAZ deletion revealed a significant decrease under semi‐rigid fixation compared to rigid (Figure 4G). Vessels in the osteotomy gap exhibited a broader orientational distribution under semi‐rigid compared to rigid fixation, both in the controls and upon endothelial YAP/TAZ deletion (Figure 4H,I). Importantly, OSX‐expressing cells were found in proximity to vessels in vascularized tissues, whereas only a few OSX‐expressing cells were found in the cartilage areas (**Figure** **5**). Therefore, EC YAP/TAZ is not required for the recruitment of OSX‐expressing cells in bone remodeling and thus for the coupling of angiogenesis and osteogenesis.

### 2.9 Fracture Fixation Determined Amount of Bone Volume in Both, Control and EC YAP/TAZ dKO Mice

In all conditions and stages of bone healing, OSX‐expressing cells were found near capillaries in the osteotomy gap. OSX is an osteoblast‐specific transcription factor required for osteoblast differentiation and bone development and is known to enhance collagen 1a2 gene expression.^[^ ^62^, ^63^ ^]^ The presence of OSX‐positive cells within collagen fiber networks indicates the capability for hydroxyapatite deposition.^[^ ^64^ ^]^ Since OSX is a determining factor for bone formation and a mediator between the coupling of osteogenesis to angiogenesis, we verified whether bone volume formed in correlation with quantities of vasculature within the osteotomy gap. Tissue mineral densities with micro‐computed tomography analyses (µCT) were assessed and bone volume changes at 14 days post‐osteotomy mice were compared between rigid and semi‐rigid fixation. As expected, more interfragmentary movement under semi‐rigid fixation led to less bone volume and thus reduced bone healing compared to rigid conditions. This was also true for EC YAP/TAZ dKO: a similar and significant reduction of bone volume was found under semi‐rigid compared to rigid fixation (Figure S6A,B, Supporting Information).

### 2.10 Endothelial YAP/TAZ Deletion led to Increased Collagen Deposition Under Semi‐Rigid Fixation

**Figure 6:**
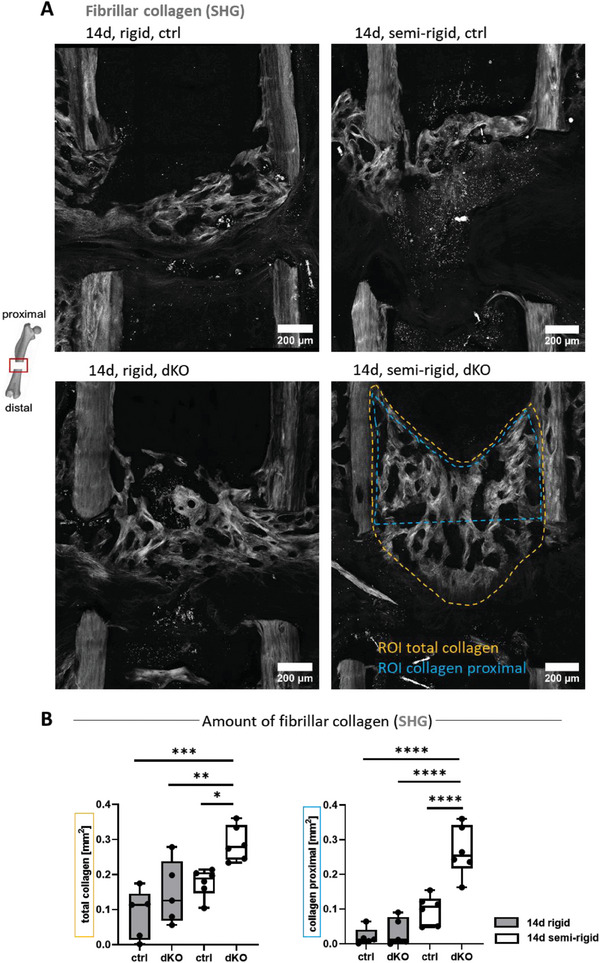
Increased collagen (SHG) under semi‐rigid fixation upon endothelial YAP/TAZ knockout compared to the control. A) Representative images of fibrillar collagen by SHG at the 14‐day time‐point. Different regions of interest, as used in our analyses, are drawn into one of the samples. B) Total collagen area quantified by SHG in the fracture gap and SHG signal proximal (ROI as defined in (A), ROI total collagen, yellow). Collagen area by SHG within the proximal bone marrow cavity (ROI as defined in (A), ROI collagen proximal, blue). Significantly increased collagen area upon endothelial YAP/TAZ knockout compared to control under semi‐rigid fixation for total collagen and collagen proximal. Statistical analysis using one‐way ANOVA followed by Tukey's test was performed. Different *p*‐values were indicated by *, **, ***, and **** for *p* < 0.05, *p* < 0.01, *p* < 0.001, and *p* < 0.0001 respectively (*N* = 5–6).

Also after 14 days, the collagen bridges connecting the proximal bone ends were still seen under rigid fixation in three out of five controls (**Figures** **6**A and S1C, Supporting Information), and upon endothelial YAP/TAZ deletion (Figures 6A and S1D, Supporting Information). Under semi‐rigid fixation, proximal collagen fiber bridges had also formed (Figure 6A, S1G), even though such bridges were not yet seen after 7 days. Again, endothelial YAP/TAZ deletion had little effect compared to the semi‐rigid control  . The collagen fibers in both cases were primarily found within the proximal bone marrow cavity (Figure S1G,H, Supporting Information), while collagen fibers were absent near the distal cortices in all cases. Quantification of total collagen fiber orientations revealed a broader distribution under semi‐rigid fixation, particularly upon endothelial YAP/TAZ deletion (Figure S7C, Supporting Information). Even though endothelial YAP/TAZ deletion had little impact on the rigidly fixed bones, total fibrillar collagen and fibrillar collagen in the bone marrow cavities were both significantly increased after deletion of endothelial YAP/TAZ under semi‐rigid fixation (Figure 6B), suggesting an effect of endothelial YAP/TAZ on surrounding matrix‐forming cells in vivo.

### 2.11 Endothelial YAP/TAZ Deletion Promoted Collagen Fiber Deposition in Surrounding Fibroblasts In Vitro

**Figure 7:**
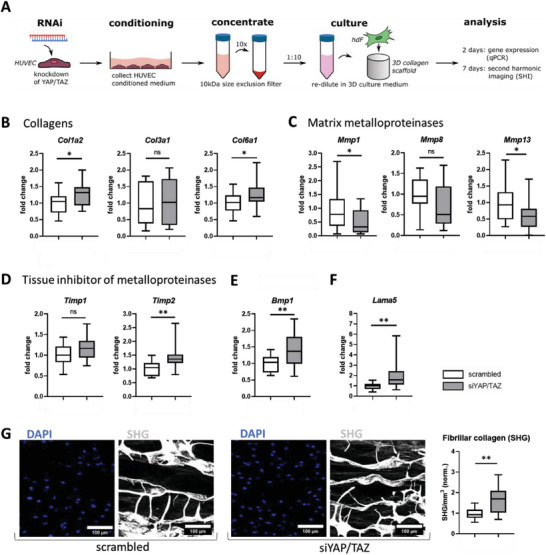
Knockdown of YAP/TAZ in HUVECs affected collagen matrix assembly and remodeling in surrounding fibroblasts through paracrine signaling. A) A transient knockdown of endothelial YAP/TAZ was performed and a conditioned medium was collected in order to treat primary human fibroblasts during cultivation within a macroporous 3D collagen scaffold. Gene expression of fibroblasts for several genes was measured 48 h after stimulation. B–F) Gene expression analysis of B) collagen type I (*Col1a2*), type 3 (*Col3a1*), and type 6 (*Col6a1*), C) matrix metalloproteinases *Mmp1*, *Mmp8*, and *Mmp13*, D) tissue inhibitor of metalloproteinases *Timp1* and *Timp2*, E) bone morphogenetic protein *Bmp1*, and F) laminin subunit alpha *Lama5*. Data are presented as fold change relative to the mean of the scrambled control. G) SHG imaging for collagen visualization was performed 7 days after stimulation. Representative images with additional DAPI staining for the scrambled and YAP/TAZ knockdown (siYAP/TAZ) are shown. Quantification of collagen signal density was performed by the cumulated signal intensity within the pores of the scaffold. Increased collagen expression of fibroblasts cultivated with conditioned media of HUVECs with YAP/TAZ knockdown compared to scrambled condition. Statistical analysis using unpaired, parametric t‐tests were performed. Different *p*‐values were indicated by * and ** for *p* < 0.05 and *p* < 0.01 compared to scrambled, respectively (*N* = 16).

Since endothelial‐specific YAP/TAZ deletion showed an increased collagen expression within the proximal bone marrow cavity upon semi‐rigid fixation, we asked whether this could at least in part be due to effects of paracrine signaling of ECs with the surrounding fibroblasts and stromal cells. To test our hypothesis, we used an in vitro approach to analyze a possible paracrine effect of endothelial YAP/TAZ signaling from HUVECs on fibroblasts. We performed a transient knockdown of endothelial YAP/TAZ in HUVECs, the efficiency of which was tested by Western blot (Figure S8, Supporting Information). A conditioned medium was collected in order to treat primary human fibroblasts during cultivation within a macroporous 3D collagen scaffold (**Figure** **7**A). A total of four experiments including the independent transfection and harvesting were performed with 4 donors each, resulting in 16 data points (*N* = 16) per condition. This scaffold has previously been shown to allow for macroscopic tissue formation and contraction through the deposition of load‐bearing collagen fibers.^[^ ^65^ ^]^ Gene expression analysis of fibroblasts was measured 48 h after stimulation. Transcript levels of various collagens revealed increased expression of collagen type 1 (*Col1a2*) and type 6 (*Col6a1*) in the YAP/TAZ knockdown condition (Figure 7B). To identify which molecular factors could be responsible for the increased collagen expression, transcript levels of selected MMPs were quantified since tissue remodeling requires MMP‐mediated ECM re‐organization.^[^ ^66^ ^]^ Indeed, collagenase *Mmp1* and *Mmp13* were slightly downregulated in fibroblasts cultured with a conditioned medium of HUVECs with transient YAP/TAZ knockdown (Figure 7C). Interestingly, this coincided with a significant upregulation of the MMP‐2 inhibitor, *Timp2* in fibroblasts, but not of *Timp1*, under the otherwise same condition (Figure 7D). Because BMP1 cleaves the C‐propeptide of type I procollagen and is thus involved in the assembly of the collagen matrix,^[^ ^67^ ^]^ we additionally checked the transcriptional level of *Bmp1*, which showed to be upregulated under the knockdown condition (Figure 7E). Finally, we also found that laminin alpha 5 *Lama5*, a key component of the basement membrane of vessels,^[^ ^68^ ^]^ was upregulated under the knockdown condition (Figure 7F). These regulations led to an increased density of collagen fibers for siYT‐treated groups compared to the controls as visualized by SHG imaging after 7 days of culture (Figure 7G). These findings suggest that endothelial YAP/TAZ, in addition to regulating target genes of their host cells, also affects their local surroundings via paracrine signaling.

## 3 Discussion

**Figure 8:**
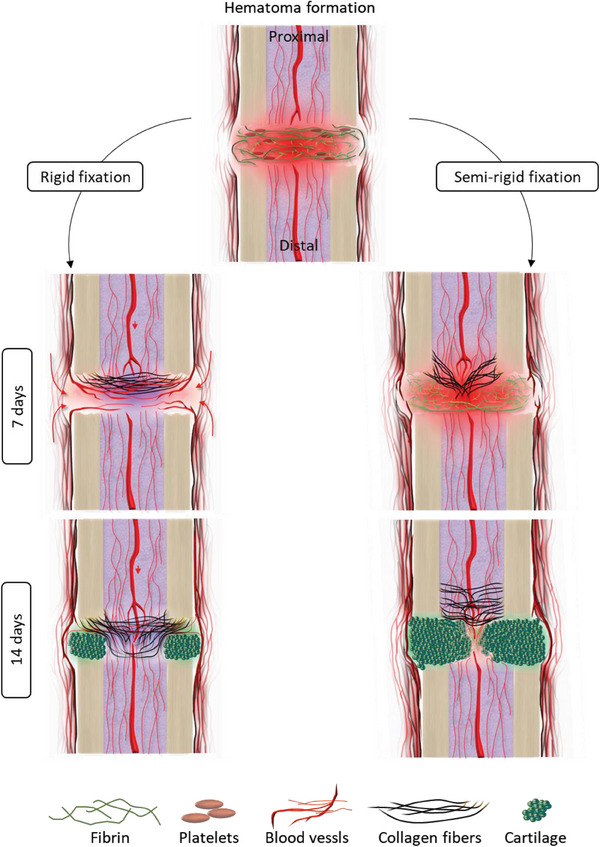
Fracture fixation stability impacts the early development of ECM and vascular architecture in bone healing, whereas endothelial YAP/TAZ activity regulates ECM‐related proteins in their surrounding environment. Bone healing initiates with hematoma formation, followed by its remodeling. We discovered that the early process of hematoma remodeling is influenced by external mechanical factors such as rigid and semi‐rigid fixation thereby dominating cellular mechanotransduction vie endothelial YAP/TAZ. At 7 days, we observed significant delays in hematoma remodeling under semi‐rigid compared to rigid fixation in both controls and upon endothelial YAP/TAZ deletion. Moreover, vessels aligned perpendicular to the central bone axis, but shifted their orientation under semi‐rigid fixation, favoring alignment parallel to the bone axis. By day 14, we observed enhanced cartilage development under semi‐rigid fixation compared to rigid even upon endothelial YAP/TAZ deletion. At 14 days, collagen fibers were predominantly oriented perpendicular to the central bone axis under rigid fixation compared to semi‐rigid fixation, in both control and dKO conditions. Hence, external mechanical stability mainly dominated local cell‐based endothelial mechanotransduction via YAP/TAZ. However, endothelial YAP/TAZ activity played a role in regulating extracellular matrix‐related proteins in their immediate environment.

This is the first investigation asking whether vascularization of a hematoma can occur even upon endothelial YAP/TAZ deletion and how this might depend on the mechanical stability of the fracture gap. Strikingly, YAP/TAZ deletion specific to ECs did not significantly alter the bone healing phases, or the sensitivity towards the mechanical stability of the fracture fixation, as quantified by analyzing the emerging morphologies of blood vessel and collagen fiber networks (Figures 1 and 2). This suggests that mechanosensation and mechanotransduction needed for sprouting angiogenesis into the hematoma is dominated by other mechanotransduction processes, including the formation of integrin adhesions to fibronectin and associated downstream signaling events. However, we found that less VE‐cadherin was recruited to stabilize cell‐cell junctions in endothelial‐specific YAP/TAZ dKO mice compared to control mice at 7 days post‐osteotomy under rigid fixation (Figure S3, Supporting Information). This is in line with previous literature showing that YAP/TAZ regulates adherens junctions during retinal vascular development.^[^ ^58^ ^]^ In contrast, the external mechanical stability of the fracture gap significantly impacted hematoma remodeling and the healing process as schematically summarized in **Figure** **8**.

Under rigid fixation, we found that the hematoma became fully vascularized (Figure 1B), whereas a less vascularized hematoma remained still visible under semi‐rigid fixation at 7 days (Figure 1C), suggesting that macroscopically imposed shear forces delayed hematoma remodeling. Under rigid fixation, the microvascular networks were adjacent to proximal collagen fibers and both were mainly oriented perpendicular to the bone cortices (Figure 1B). Previously it has been shown that collagen fibers form a shell‐like structure closing the marrow cavity in early bone healing in rats.^[^ ^69^ ^]^ This is striking as vessels and collagen fibers are aligned parallel to the bone axis in a completely healed bone. This raises the question of why “natural” bone healing starts out with a perpendicular orientation in the early phase. This may result from early ECM fiber organization which is steered by the contractile activity of blood clot entrapped platelets.^[^ ^70^, ^71^ ^]^ Upon their contraction and through the force that platelets exert on the fibrin fibers, they organize their surrounding fibrin matrix mediated via αIIbβ3 integrin binding to fibrinogen.^[^ ^15^, ^72^, ^73^ ^]^ As it is well known that cell‐contracted isotropic fiber networks become anisotropically aligned with respect to the positions of the external anchor points,^[^ ^74^ ^]^ we thus suggest that the proximal bone tips might serve as primary anchor points for the fibrin networks. The isotropic fibrin network that had filled the fracture gap, hence becomes anisotropic upon platelet contraction, thereby aligning the fibers perpendicular to the cortices. Platelets also deposit fibronectin fibers^[^ ^15^ ^]^ that might subsequently serve as the first templates to nucleate collagen fibrillogenesis,^[^ ^75^ ^]^ upon secretion of collagen by invading MSCs and fibroblasts.^[^ ^10^ ^]^ Our data suggest that in the early bone healing phase, provisional early fibrin and fibronectin fiber organization determines the structural organization of the collagen and of the microvascular networks. We propose that ECs during sprouting angiogenesis then migrate and align along those ECM fibers thereby directing the vascular networks which is in accordance with a previous study demonstrating the migration of ECs along fibronectin fibers in vitro.^[^ ^76^ ^]^ Decreased mechanical stability under semi‐rigid fixation though resulted in collagen fibers oriented more parallel to the cortices on the proximal side (Figure 1C). Possibly, increased interfragmentary movement together with enhanced fluid flow might lead to breakage of early collagen bridges connecting the proximal bone ends, which could lead to a switch of the collagen fiber orientations. Moreover, collagen deposition was mainly found within the proximal bone marrow cavity (Figure 1C and Figure S1E, Supporting Information), which is the site of the least strain. Concomitantly, microvascular networks mainly formed in collagen‐rich regions (Figure 1C), again suggesting a close interdependency of ECs and fibrillar collagen. In contrast, SHG‐positive collagen fibers had not yet emerged on the distal side on day 7, however, our data suggest that EC sprouting and vascularization might be guided also on the distal side by anisotropic fibrin or fibronectin fibers under rigid fixation.

Semi‐rigid fixation significantly delayed the vascularization of the gap at day 7, and the hematoma was still present, particularly pronounced at the distal side (Figure 1C). Based on the lack of blood vessels, it was typically assumed that these areas are hypoxic,^[^ ^44^, ^77^ ^]^ yet in contradiction with this assumption, hypoxia is known to promote the expression of hypoxia‐inducible factor‐1α (HIF‐1α), and thus angiogenesis.^[^ ^78^ ^‐–^ ^82^ ^]^ As the cells entrapped in the blood clot^[^ ^10^, ^83^ ^]^ already require a substantial supply of nutrients to survive, and play a major role in the hematoma remodeling, we suggest an alternative to explain the delayed hematoma vascularization under semi‐rigid fixation. Under rigid fixation, the oxygen demand may indeed lead to hypoxic conditions which then promote the early vascularization of the fracture gap. Under semi‐rigid fixation though, oxygen transport could be upregulated by mass transport as driven by local compression and shear as the animal moves, as the local movement of the hematoma sucks in, and presses out the surrounding fluid. We propose that shear‐enhanced mass transport within the osteotomy gap under semi‐rigid fixation enhances oxygen availability in those tissues in motion, which is expected to enhance oxygen supply compared to rigid fixation in the absence of vessels. As in vitro studies suggested that the blood clot entrapped cells do not yet express VEGF, but only in synergy with invading mesenchymal cells,^[^ ^10^, ^83^ ^]^ reduced number of invading cells under semi‐rigid versus rigid fixation could further explain the delayed hematoma vascularization. Movat's pentachrome staining confirmed that some hematoma‐filled regions were still visible on the distal side adjacent to the bone marrow even after 14 days post‐osteotomy (Figure S5, Supporting Information). This is in agreement with sheep studies which showed that mRNA expression levels of vWF, Ang1, Ang2, VEGF, CYR61, FGF2, MMP2, and TIMP1 were distinctly lower in the delayed compared to the rigidly‐fixed group at several time points.^[^ ^44^ ^]^ Finally, low oxygen tension conditions are more potent promoters of chondrogenic differentiation than dynamic compression,^[^ ^84^ ^]^ which can further explain why we see a delayed onset of chondrogenesis on the distal side under semi‐rigid compared to rigid fixation. Taken together, our hypothesis that mass transport plays a major role in the oxygenation of the hematoma under semi‐rigid fixation is in agreement with observations from us and the literature.

In the next stage of fracture healing, chondrocyte differentiation and cartilage development could be facilitated by multipotent MSCs that invade the osteotomy gap from the periosteum and bone marrow and can differentiate towards osteoblasts or chondrocytes.^[^ ^85^ ^]^ Recent evidence suggests though that the hematoma itself may offer a source of cells with chondrogenic potential, as hematoma‐derived cells can differentiate into hypertrophic chondrocytes and induce subsequent calcification of the ECM in vitro.^[^ ^86^ ^]^ Hence, we would like to propose that the cartilage tissue that developed in the healing region (Figure 4A–E, and Figures S4 and S5, Supporting Information) could be at least partially derived from the hematoma itself and not from invading MSCs, even though some MSC invasion cannot be excluded. Further studies would be needed to verify whether and to what extent hematoma‐derived cells could indeed be a source for chondrogenesis in vivo.

Finally, our images revealed that collagen fiber deposition within the proximal bone marrow cavity under semi‐rigid fixation was upregulated in EC YAP/TAZ dKO mice compared to their controls at 14 days (Figure 6B). This tendency could already be observed at the 7‐day time point (Figure S1E,F, Supporting Information). Together with our in vitro data, where HUVEC's supernatant (scrambled and siYAP/TAZ) was used for 3D fibroblast cell culture (Figure 7), these findings could be explained by the following: Nuclear YAP/TAZ was shown to suppress endothelial BMP expression in the retina, and vice versa, YAP/TAZ deletion upregulated BMP signaling.^[^ ^58^ ^]^ Since BMP1 promotes cleavage of procollagen and thus osteogenesis,^[^ ^87^ ^]^ this could enhance collagen fiber deposition by the YAP/TAZ depleted ECs directly, or by the surrounding mesenchymal cells via paracrine BMP signaling, ultimately suggesting an explanation for the increased collagen deposition in EC YAP/TAZ dKO mice compared to the controls. The reason why semi‐rigid fixation seems to enhance this effect needs to be explored in future studies.

Taken together, our work illustrates that sprouting angiogenesis into a freshly formed hematoma and its vascularization does not require the presence of endothelial YAP/TAZ, and thus the expression of genes regulated by the nuclear translocation of YAP/TAZ. Instead, the vascular morphology as well as the orientation of collagen fibers during the healing process is mostly controlled by the stability of the fracture fixation. This understanding of the important role of fracture stability compared to endothelial YAP/TAZ directs future research to unravel the specific mechanisms through which stability influences healing. Next to direct clinical consequences on how to stabilize fractures, delving into these mechanisms might uncover specific signaling pathways or molecular interactions that can be modulated to optimize fracture healing. This may open new avenues for innovative therapeutic approaches to bone regeneration, even when surgical fixation lacks optimized stability.

## 4 Experimental Section

### Mouse Lines for Endothelial Specific YAP/TAZ Deletion

For loss of function experiments, the *Yap^fl/fl^ Taz^fl/fl^*,^[^ ^48^ ^]^ *Cdh5‐iCreERT2* (Tg(Cdh5‐cre/ERT2)1Rha)^[^ ^49^ ^]^ mouse strain was used. All animals were homozygotes for the Wwtr1 and Yap1 genes. The Cdh5‐Cre‐allele was heterozygote in the animals where Yap/Taz was silenced by tamoxifen, the controls in this study were littermates lacking the Cdh5‐Cre‐allele. All animals were age‐ and sex‐matched. Mice were maintained at the Max Delbrueck Center for Molecular Medicine under standard husbandry conditions. To induce Cre‐mediated recombination, tamoxifen (Sigma‐Aldrich #T5648) was used. In order to avoid bias from the tamoxifen treatment, all animals (test and control) were equally injected with tamoxifen three times intraperitoneally at the age of 8 weeks (200 g pro kg per day, every second day). The animals were randomly distributed in the rigid or semi‐rigid treatment groups and sample processing was blinded until data evaluation was performed. Operations were performed at the Julius Wolff Institute of Biomechanics and Musculoskeletal Regeneration and femurs were collected 7 or 14 days later (LaGeSo G0322/18). For recombination efficiency, genomic DNA from bone marrow and growth plate samples was used (NucleoSpin DNA Forensic Kit from Macherey and Nagel, #740840.50) and genotyping PCRs were performed as described in Gruber et al., 2016,^[^ ^48^ ^]^ using the following PCR primers for Yap:Yap1‐delta‐f 5′‐CACAGAGATCCTCCTGTCTCAG‐3′ and Yap1‐r 5′‐TGTTGTCATATGCCATTGTGTAA‐3′ producing PCR band of 457 bp (Yap‐ recombined, Δ) and PCR primers used for Taz/Wwtr1 genotyping were Taz‐f 5′‐AGCAAAGTAAGGGCACTGTATG‐3′, and Taz‐delta‐r 5′‐TCTACTCTTGGCTCTTAGCTGG‐3′ producing PCR band of 249 bp (Taz‐ recombined, Δ) (HotStarTaq *Plus* Master Mix Kit, Qiagen #203645).

### Mouse Osteotomy Model to Study Bone Regeneration

The fixator assembly and osteotomy were performed under isoflurane anesthesia. The anesthesia was maintained via a mask with an isoflurane‐oxygen mixture. Animals received a subcutaneous injection of buprenorphine (Temgesic) (0.03 mg kg^−1^) while still under anesthesia before surgery. Prophylactic antibiosis using clindamycin (8 mg kg^−1^ KG) was administered preoperatively, while the animal was still anesthetized, to prevent intraoperative contamination. Surgery was performed on a warming plate to prevent chilling of the animals during the procedure. The eyes of the mice were protected from drying out by eye ointment during each anesthetic. An external fixator was mounted on the left femur of the animals under general anesthesia. This commercially available fixator (MouseExFix 100% and 50%, RISystem) consisted of four screws (Ø = 0.45 mm) and a radiolucent fixator body. A lateral longitudinal skin incision was made from the knee to the hip joint. After dissection of the iliotibial tract and vastus lateralis, the femur was exposed while sparing the sciatic nerve. The first drill hole for pin insertion was drilled just proximal to the distal metaphysis of the femur using a hand drill. This was followed by drilling of the other three pinholes for attachment of the external fixator. After the application of the external fixator strictly parallel to the femoral axis, a 0.7 mm osteotomy was then performed with a wire saw (RISystem, Davos, Switzerland) between the two middle pins. The wound was closed in layers. Subsequently, the animals received tramadol via drinking water (25 mg L^−1^) for 3 days postoperatively. The mice were able to move in the cages with the fixators in place as their healing progressed. Animals were sacrificed 7 and 14 days post‐osteotomy under deep anesthesia with a mixture of ketamine and medetomidine by cervical dislocation.

### Rigid and Semi‐Rigid Fixators as a Model System for Successful and Delayed Healing

A previously established external fixation system was employed here that allowed for control of the local mechanical setting at a fracture site. The system was fully described earlier^[^ ^42^, ^88^ ^]^ and was briefly summarized: An external fixator of the company RISystem (RISystem AG, Davos, Switzerland) was employed that allowed for a rigid fixation of fracture fragments (summarized as “rigid fixation”) that will lead to a 0.5 mm osteotomy gap to heal within 3 weeks to the contralateral intact bone mechanical properties. If the fixator was however equipped with a semi‐rigid fixation bar, the shear movements were doubled and the tissue shear straining substantially increased, resulting in a substantial delay in healing. The tissue strainings were quantified for both systems, rigid and semi‐rigid fixations, using finite element analyses (FEA) techniques in a previously developed and validated model of a 0.5 mm osteotomy gap.^[^ ^89^ ^]^ This model was adapted to a 0.7 mm osteotomy gap as used in the current study. At the 7‐day time point, the adapted model predicted the average absolute maximum principal strains within the 0.7 mm osteotomy gap to be −11.1 ± 0.9% in the case of rigid fixators and −44.8 ± 3.9% for semi‐rigid fixators.^[^ ^90^ ^]^ During the time course of healing, the onset of endochondral ossification as well as of the later mineralization phases led to a reduction of the initial mechanical straining until bone bridging was reached.

### Bone Sample Preparation and Micro‐Computed Tomography

After mice were sacrificed, the femurs were harvested and incubated in 4% PFA in PBS for 6–8 h on a shaker. Femurs were then loaded on a custom‐made sample holder and scanned with a Bruker SkyScan 1172 high‐resolution µCT (Bruker, Kontich, Belgium). Parameters measured include mineralized callus volume (BV, mm^3^), total callus volume (TV, mm^3^), fraction of mineralized callus volume (BV/TV), and bone mineral density of callus tissue (BMD, mg HA cm^−3^). After the scan bone samples were washed with PBS and for further processing transferred to 0.5 M EDTA in H_2_O for decalcification and incubated for 24 h at 4 °C on a shaker. After the removal of EDTA solution, sucrose solution (20% sucrose/ 2% PVP/H_2_O) was added and incubated for 6 h at 4 °C on a shaker. Bones were transferred into a cryosection mold and mounted with embedding bone medium (8% gelatin/ 20% sucrose/ 2% PVP in H_2_O). Bones were stored at −80 °C. Cryosections were prepared for immunostaining and imaging.

### Cryosectioning

Cryoblocks were mounted on a cryotome sample holder using a Tissue‐Tek O.C.T. Compound (Sakura, # 4583). 50 µm thick sections were cut and directly transferred to glass slides. 7 µm thick sections were first transferred to Kawamoto tape (Section Lab, # C‐FP096) and then transferred to a glass slide and fixed with tesafilm strips. The thick sections were used for immunostaining, whereas thin sections were used for Movat's pentachrome staining.

### Immunofluorescence Staining

Tissue cryosections were thawed and dried for 30 min at room temperature. After rehydration with ice‐cold PBS for 5 min, samples were permeabilized with ice‐cold 0.3% triton in water for 10 min. Samples were then blocked with 5% normal serum donkey and 0.3% triton in 1× PBS for 30 min. Primary antibodies against monoclonal rat anti‐endomucin (Santa Cruz, Cat# sc‐65495, 1:100), or polyclonal goat anti‐CD31/PECAM‐1 (R&D systems, #AF3628, 1:200), polyclonal rabbit anti‐osterix/Sp7 (Abcam, ab22552, 1:200), or monoclonal rabbit anti‐VE‐cadherin (Abcam, [EPR18229] ab205336, 1:200) were diluted in 5% normal serum donkey in PBS with their respective dilution and incubated overnight at 4 °C. Samples were then washed with ice‐cold PBS three times for 5 min. Secondary antibodies (Alexa 546, A11056, Invitrogen; Alexa 488, A21206, Invitrogen; Alexa 647, A31573, Invitrogen) were diluted in PBS with a 1:200 dilution. For some tissue sections, DAPI was added as well. Samples were then incubated with a secondary antibody solution for 3 h before washing with PBS was performed. Samples were then mounted with Fluoromount G (#Cat 0100–01, Southern Biotech).

### Vessel and Platelet Visualization by Two Distinct Markers: CD31/PECAM‐1 and EMCN

CD31, also called PECAM‐1, is a cell‐cell adhesion protein and expressed on the surfaces of arterial and venous ECs, but also of platelets and immune cells.^[^ ^91^ ^]^ EMCN, an O‐glycosylated single transmembrane sialomucin, is expressed by capillary and venous ECs^[^ ^38^ ^]^ and regulates angiogenesis by controlling VEGFR2 signaling.^[^ ^92^ ^]^ Immune‐histological staining was performed with these markers. The protocol followed for CD31 and EMCN staining was established previously.^[^ ^93^ ^]^

### Imaging of Histological Tissue Sections

Images were acquired with a Leica SP5 confocal microscope. 25× water immersion objective for overview images and 40× water immersion objective for zoomed‐in images were used. Fibrillar collagen was visualized by SHG imaging using a multiphoton laser with excitation at 910 nm. Z‐stacks were acquired for every image.

### Image Analysis and Quantifications

Acquired images were further analyzed with Fiji using maximum intensity projections of acquired image stacks. For vessel quantification, the quantity of EMCN‐positive vessels within the osteotomy gap was measured relative to the total osteotomy gap area. To assess vessel orientation, images of EMCN‐positive vessels were transformed into skeletonized binary images. The orientations of the vessels within the marked ROIs distinguishing between the osteotomy gap and proximal bone marrow were then assessed using a custom macro implemented in Fiji. The percentage of orientation distribution was then plotted in a polar diagram with Origin Pro2021b representing the mean value of all samples analyzed and the standard deviation. Additionally, the mean vessel diameters (µm) in defined ROIs within both the proximal and distal bone marrow were calculated using a custom macro and Fiji. A detailed schematic can be found in Figure S9, Supporting Information. The quantification of the amount of fibrillar collagen was analyzed from SHG images using Fiji and a custom macro. A threshold was set to identify SHG‐positive pixels while excluding SHG‐positive signals originating from the cortices. SHG‐positive tissue area (mm^2^) was measured within both the proximal bone marrow cavity and the osteotomy gap. The orientation of fibrillar collagen was analyzed using SHG images with the ImageJ plug‐in FibrilTool.^[^ ^94^ ^]^ Briefly, ROIs were manually selected to capture the SHG‐positive areas within the osteotomy gap and proximal marrow cavity. Cortices were excluded from the analysis. Each ROI was divided into sub‐ROIs, with the average orientation within each sub‐ROI depicted as a red line. A distribution plot of the average collagen fiber orientation of all sub‐ROIs of all analyzed samples was generated using Origin Pro2021b.

### Cartilage Quantification

To assess the histomorphometric quantification of cartilage area, Movat pentachrome images were captured utilizing the Zeiss Axioskop 40 bright‐field microscope. Digital image analysis was conducted using Fiji, along with a custom macro, which enabled the quantification of the cartilage area within a specified ROI. Cartilage fractions (%) were then calculated based on the defined ROI.

### siRNA Knockdown and Conditioned Medium Harvesting

Primary human umbilical vein endothelial cells (HUVECs) were used between passages P3 and P5. Cells were cultured in EGM‐2 medium (Lonza, CC‐3162). Cells were transfected using Dharmafect (Dharmacon, T‐2001‐03) and siRNAs for TAZ (Dharmacon, M‐016083‐00‐0020) and YAP (M‐012200‐00‐0020) and siControl (Dharmacon, D‐001206‐13‐20) at a concentration of 25 nM according to the manufacturer's instructions. 24 h after transfection, the transfection medium was replaced by fresh EGM‐2 containing 1% FBS and including supplements. Conditioned media were collected after an incubation period of an additional 48 h and processed according to a protocol described previously.^[^ ^95^ ^]^ In brief, conditioned media were centrifuged at 700 × g for 8 min to remove debris and then passed through a 0.2 µm sterile filter. Amicon ultra centrifugation units (10 kDa, Merck, UFC901024) were used to concentrate media to 10×. Concentrates were re‐diluted to 1× during further culture.

### In Vitro 3D Culture and Imaging

Primary human skin biopsies (*n* = 4, age 24–51 years) were provided by the BCRT Core Unit “Cell and Tissue Harvesting”. Patient recruitment, sample harvest, and fibroblast isolation were approved by the Institutional Review Board of the Charité and all patients gave their written informed consent. Primary human dermal fibroblasts were cultured as described previously.^[^ ^65^ ^]^ In brief, primary fibroblasts were isolated from skin biopsies by outgrowth culture. Cells were cultivated in DMEM (Thermo Fischer, 11960‐044), supplemented with 10% fetal bovine serum (FBS, Sigma Aldrich, S0615), 1% penicillin/streptomycin (P/S, Bio&Sell, BS.A 2212), and 1% non‐essential amino‐acids (NEA, Bio&Sell, BS. K0293) under 5% CO2 in a humidified incubator. Cells were used between passages 4 and 6 and sub‐cultivated when reaching confluency using 1× Trypsin‐EDTA solution.

Cylindrical, 5mmØ, macroporous 3D collagen scaffolds (*E* = 6.5 kPa Matricel GmbH) were seeded with primary human fibroblasts at a concentration of 7500 cells per mm^3^ by dip‐in update of a concentrated cell suspension. Cells were allowed to adhere at 37 °C for 1 h without additional medium and then cultured in expansion medium overnight prior to stimulation with conditioned medium.

Concentrated conditioned media were re‐diluted to 1× in ECM formation media consisting of DMEM supplemented with 1% P/S, 1% NEA, 2% FBS, and 1.36 mM ascorbic acid. The medium was replaced after 3 days once. Samples were fixed with 4% paraformaldehyde and the reaction was quenched with 25 mM NH_4_Cl/PBS for at least 1 h. Fixed samples were embedded in 5% gelatin/PBS solution to allow cutting into cylinder halves using a scalpel. Cylinder halves were further processed using a cryotome (Leica) to prepare a smooth surface for confocal imaging. After cryo‐cutting, actin and nuclei staining were performed according to manufacturer's instructions (Phalloidin Atto 550, Merck, 19083; Sytox‐Deep red, Thermo Fischer, S11381) and images were acquired via confocal imaging using a Leica SP5 inverted microscope equipped with a MaiTai Multiphoton Laser (SpectraPhysics) and a 25× water immersion objective. Images were acquired with a resolution of 1.22 × 1.22 × 4 µm (*xyz*) voxel size. Quantification of collagen signal density was performed by the cumulated signal intensity within the pores of the scaffold.

### Gene Expression Analysis

RNA was isolated using the PureLink RNA Mini Kit (Thermo Fischer, 12183018A) according to the manufacturer's instructions in combination with PureLink DNase (Thermo Fischer, 12185010) to digest genomic DNA. cDNA synthesis was performed using the iScript cDNA Synthesis Kit (Bio‐Rad, #170‐8891) according to the manufacturer's instructions. Quantitative PCR was performed using iQ5 Real‐Time PCR Detection System (Bio‐Rad) in combination with Luna qPCR SybrGreen master mix (NEB, M3003). Mean normalized expression levels were calculated according to the ΔC_T_‐method^[^ ^96^ ^]^ with primer efficacy correction as described previously^[^ ^97^ ^]^ and fold changes were calculated by normalizing MNE values to the mean siScrambled value. Hypoxanthine‐guanine phosphoribosyl transferase 1 (HPRT1) was used as a reference gene. All primer sequences are summarized in Table S1, Supporting Information.

### Western Blot

For Western blots, the protein was extracted using an M‐PER protein extraction buffer and a Halt protease and phosphatase inhibitor cocktail (Thermo Fischer Scientific, 78441). Protein concentration was determined using the BCA protein assay kit (Pierce, 23227). Protein was separated by SDS‐PAGE. Protein was transferred to a blotting membrane (Amersham, 10600023). The membrane was stained with primary antibodies 1:500 YAP (Invitrogen, PA1‐46189), 1:500 TAZ (Sigma‐Aldrich, HPA007415), 1:4000 GAPDH (MAB374, Millipore) overnight at 4 °C and secondary antibodies coupled with HRP (Horseradish peroxidase), at a concentration of 1:5000 for 1 h at RT. Protein bands were visualized using an ECL kit (Amersham, RPN2232) and detected using an imaging device (ImageQuant 800, Amersham).

### Statistics

Using common test approaches and laboratory conditions and assuming realistic error sizes and case numbers while accepting conservative errors type I and II of 0.05 and 0.2 and using non‐parametric test procedures, a group size of *N* = 6 was determined that allowed the detection of effect sizes between 1.5 and 1.8. The legal authorities allowed the authors, based on the above power calculation, to have *N* = 6 max per group in the experiments. The number of animals (“N”) included in each of the postprocessing steps was indicated in detail across the whole manuscript.

Two‐tailed paired *t*‐tests were performed with GraphPad Prism software to test for statistical significance between the two groups. Comparisons between four groups were made using one‐way ANOVA followed by Tukey's test. Different *p*‐values were indicated by stars in corresponding figures (*p < 0.05, **p < 0.01, ***p < 0.001, ****p < 0.0001).

## Conflict of Interest

The authors declare no conflict of interest.

## Supporting information

Supporting Information

## Data Availability

The data that support the findings of this study are available from the corresponding author upon reasonable request.
