# Supplementary material for: External Mechanical Stability Regulates Hematoma Vascularization in Bone Healing Rather than Endothelial YAP/TAZ Mechanotransduction
Source: Adv Sci (Weinh). 2024 Jan 25;11(13):2307050. doi: 10.1002/advs.202307050 (PMC10987120; doi:10.1002/advs.202307050)
Supplement: Supplementary file 1 — Supporting Information [file ADVS-11-2307050-s001.pdf]

ADVANCED SCIENCE
Open Access

# Supporting Information

for *Adv. Sci.*, DOI 10.1002/advs.202307050

External Mechanical Stability Regulates Hematoma Vascularization in Bone Healing Rather than Endothelial YAP/TAZ Mechanotransduction

*Julia Mehl, Saeed Khomeijani Farahani, Erik Brauer, Alexandra Klaus-Bergmann, Tobias Thiele, Agnes Ellinghaus, Eireen Bartels-Klein, Katharina Koch, Katharina Schmidt-Bleek, Ansgar Petersen, Holger Gerhardt*, Viola Vogel* and Georg N. Duda**

# Supporting information

**External mechanical stability regulates hematoma vascularization in bone healing rather than endothelial YAP/TAZ mechanotransduction**

*Julia Mehl, Saeed Khomeijani Farahani, Erik Brauer, Alexandra Klaus-Bergmann, Tobias Thiele, Agnes Ellinghaus, Eireen Bartels-Klein, Katharina Koch, Katharina Schmidt-Bleek, Ansgar Petersen, Holger Gerhardt*, Viola Vogel*, Georg N. Duda**

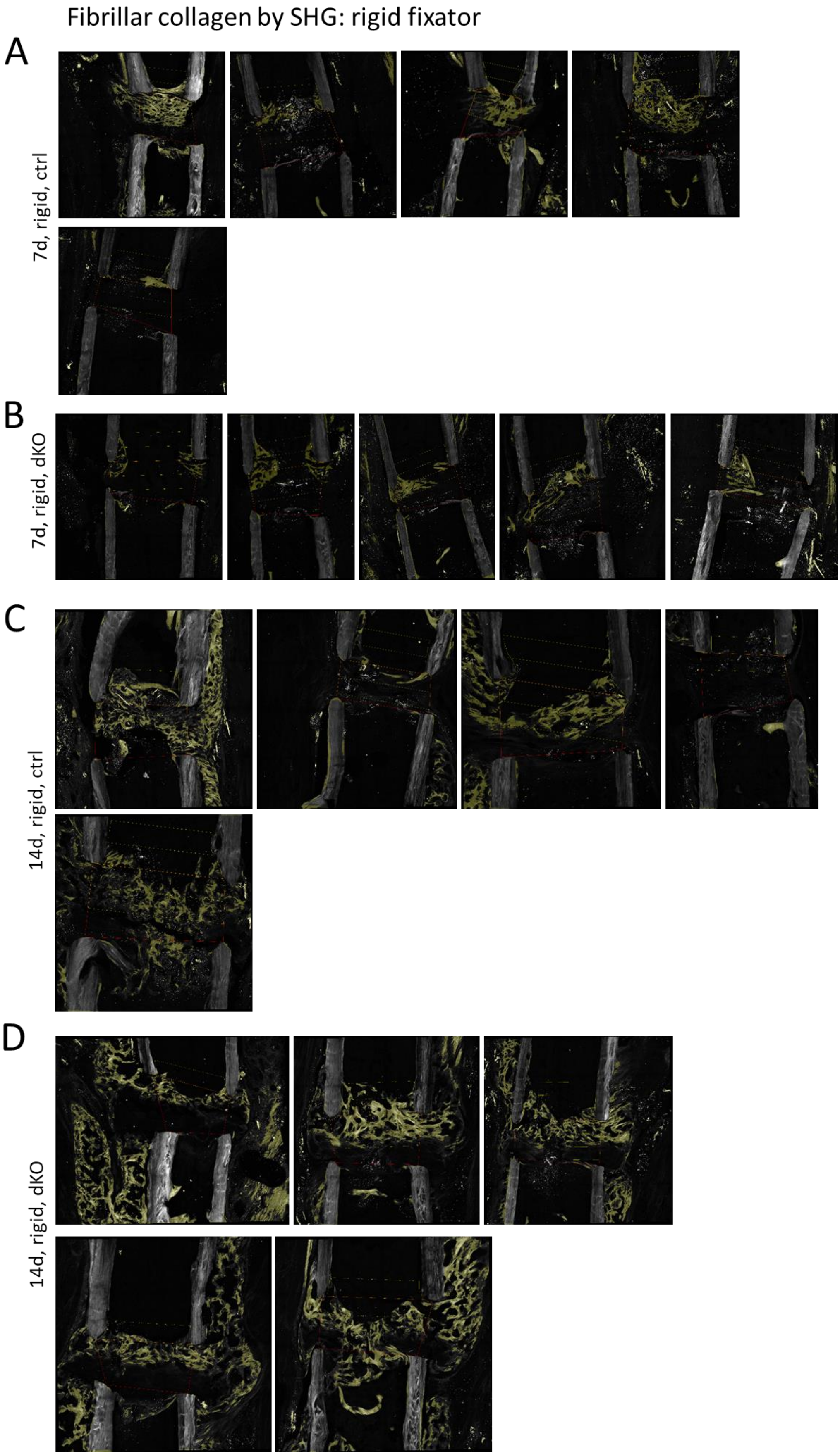
Fibrillar collagen by SHG: rigid fixator
A
7d, rigid, ctrl
B
7d, rigid, dKO
C
14d, rigid, ctrl
D
14d, rigid, dKO

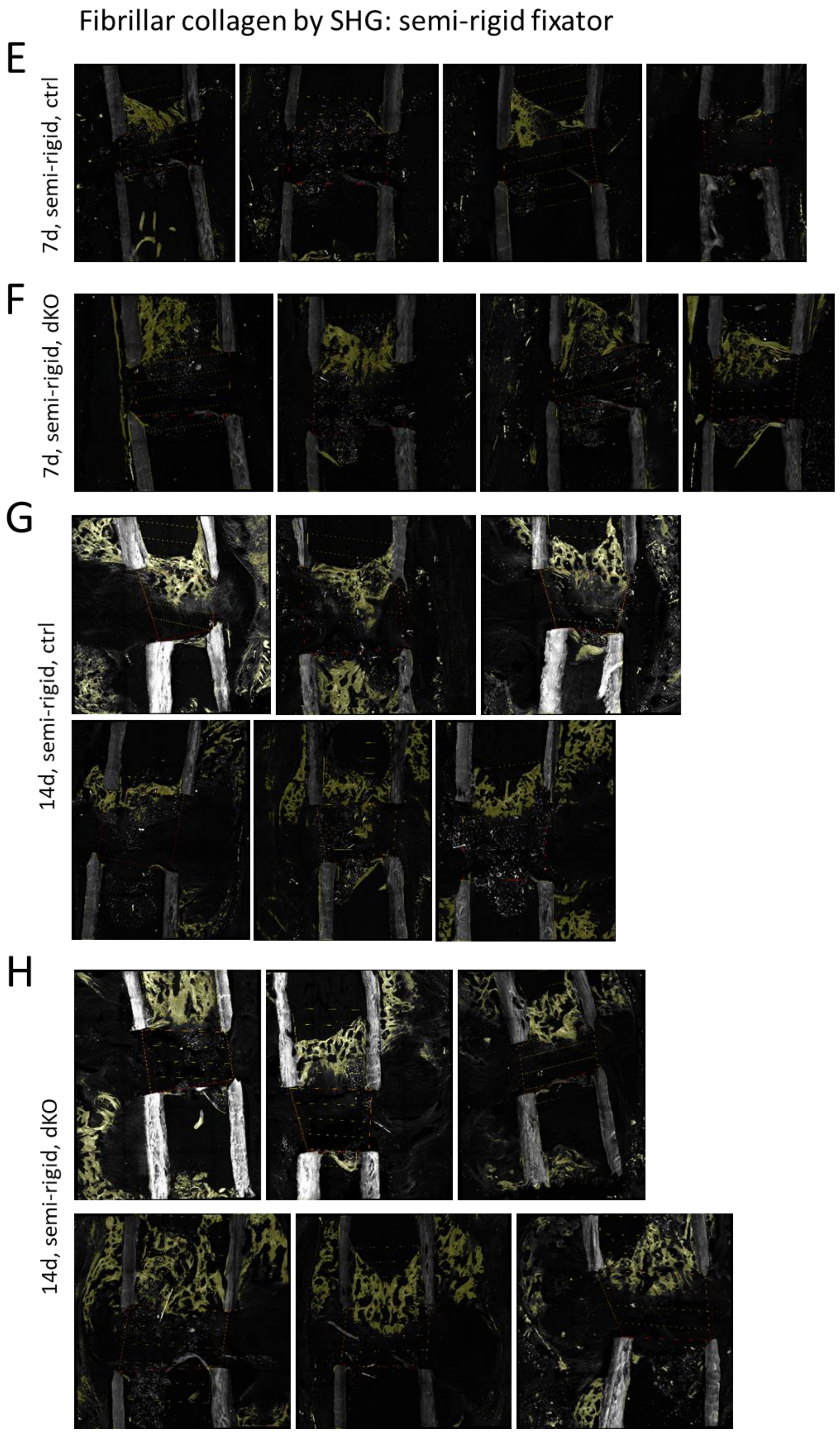


**Figure S1. Fibrillar collagen as quantified by SHG.** Images are shown for all mice used in this study under the different fixation conditions as indicated **(A-H)**. Collagen fibers were visualized by SHG imaging. The greenish area in the osteotomy gap and proximal bone marrow

cavity indicates the SHG signal identified by our macro and used for quantifications. The SHG signal from the cortical fracture ends and the periosteal SHG signal were not considered for the quantifications.

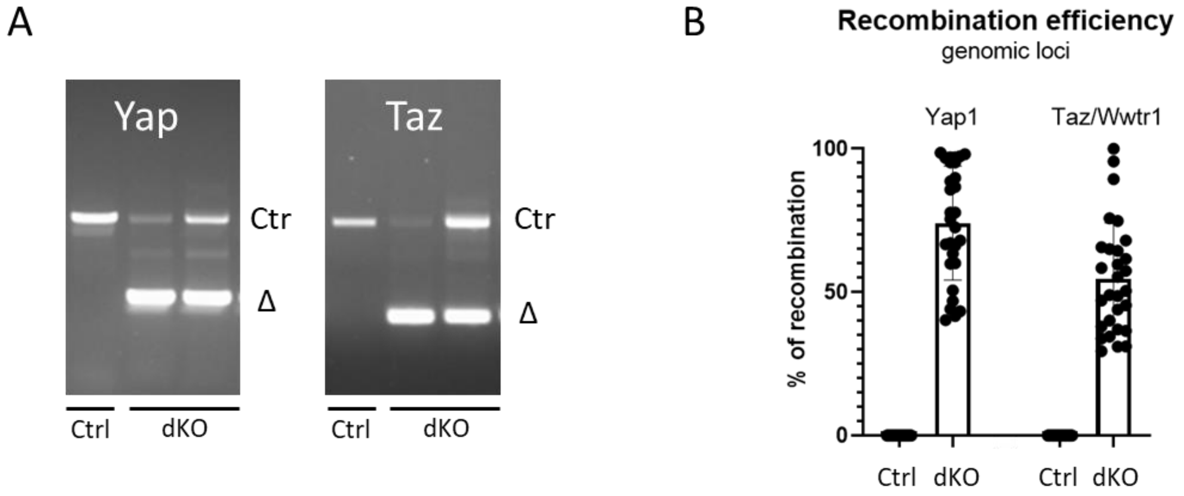


**Figure S2. Recombination efficiency in control and endothelial YAP/TAZ dKO mice**. **A.** PCR genotyping shows conditional deletion in the *Yap1* and *Taz/Wwtr1* gene of bone marrow and growth plate samples. **B.** Quantification of recombination efficiency are shown as the percentage of recombined (Δ) vs Ctrl PCR band intensities.

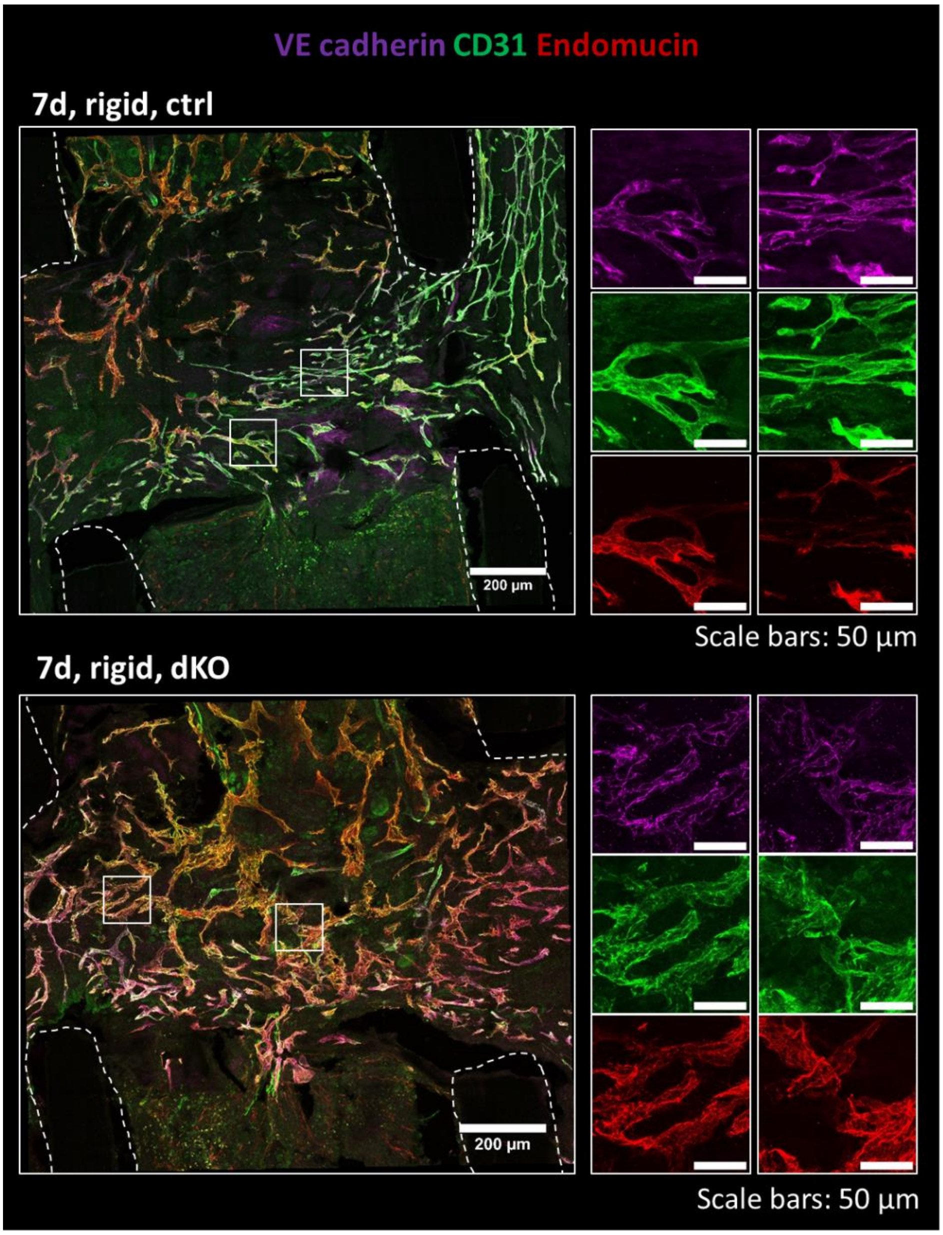


**Figure S3**. **Reduced VE-cadherin in adherens junctions upon endothelial specific YAP/TAZ deletion in the osteotomy gap compared to the control under rigid fixation.** Immunofluorescence staining was performed for EMCN and CD31 for vessel visualization. VE cadherin was stained to visualize EC-EC adherens junctions. Representative images for control and EC YAP/TAZ dKO mice at 7 days post-osteotomy show significantly more faint VE cadherin junctions in EC YAP/TAZ dKO mice compared to control. Despite the fact, that in both, control and EC YAP/TAZ dKO, the gap was fully vascularized in early bone healing, newly forming vessels in the osteotomy gap required at least in part endothelial YAP/TAZ for the adherens junctions to mature. Concomitantly, vessel markers CD31 and EMCN were more faint upon endothelial YAP/TAZ deletion compared to control (scale bars = 200 µm; zoom-in images = 50 µm).

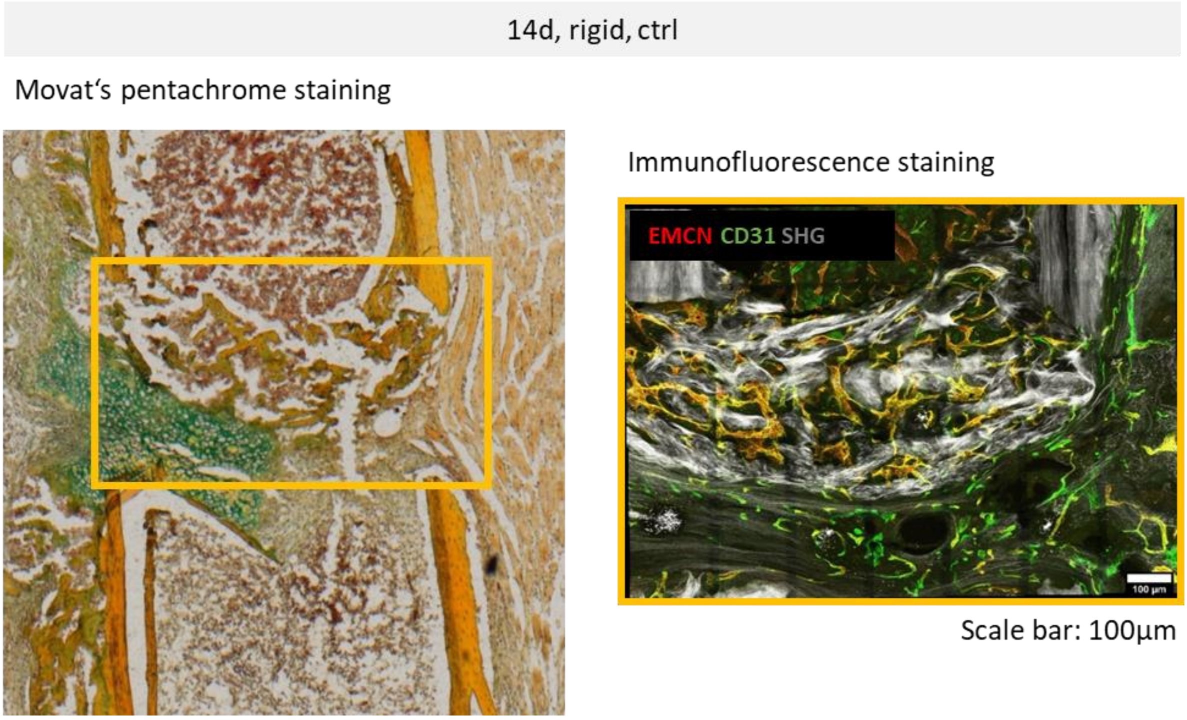


**Figure S4. Movat’s pentachrome staining confirmed the presence of cartilage**. Cartilage developed in the osteotomy gap 14 days post-osteotomy upon rigid fixation as confirmed by the greenish area at the Movat pentachrome image. The zoom-in image on the right side shows the corresponding immunofluorescence image illustrating the vanishing of vessels in regions of cartilage development.

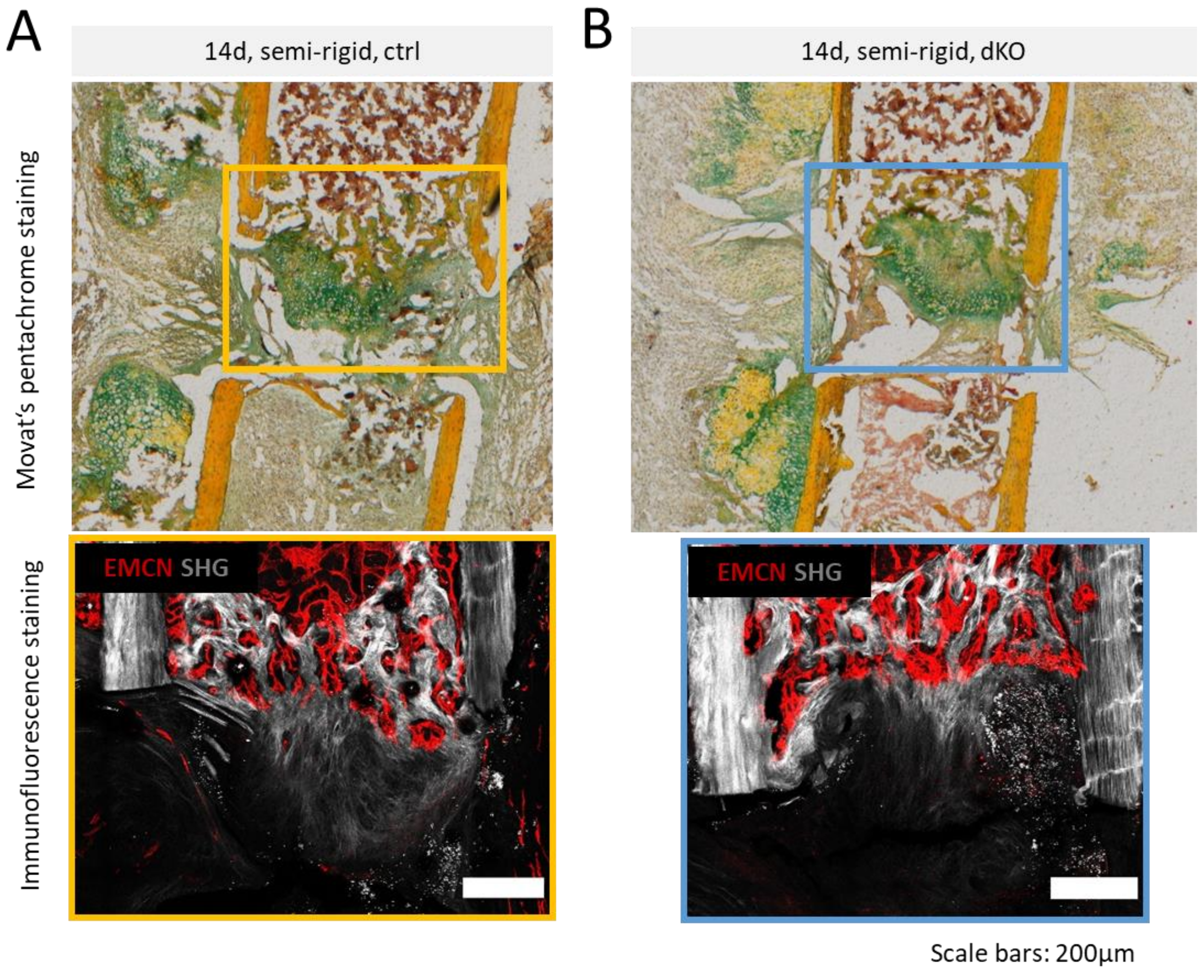


**Figure S5. Movat's pentachrome staining shows that even more cartilage developed in semi-rigid compared to rigid, in both, control and EC YAP/TAZ dKO mice. A.** Movat's pentachrome staining for control, semi-rigid. The zoom-in image shows corresponding immunofluorescence image of EMCN superimposed with SHG signal. Cartilage development started directly beneath the vascular front. **B.** Cartilage development in endothelial specific YAP/TAZ deleted mice similar to control.

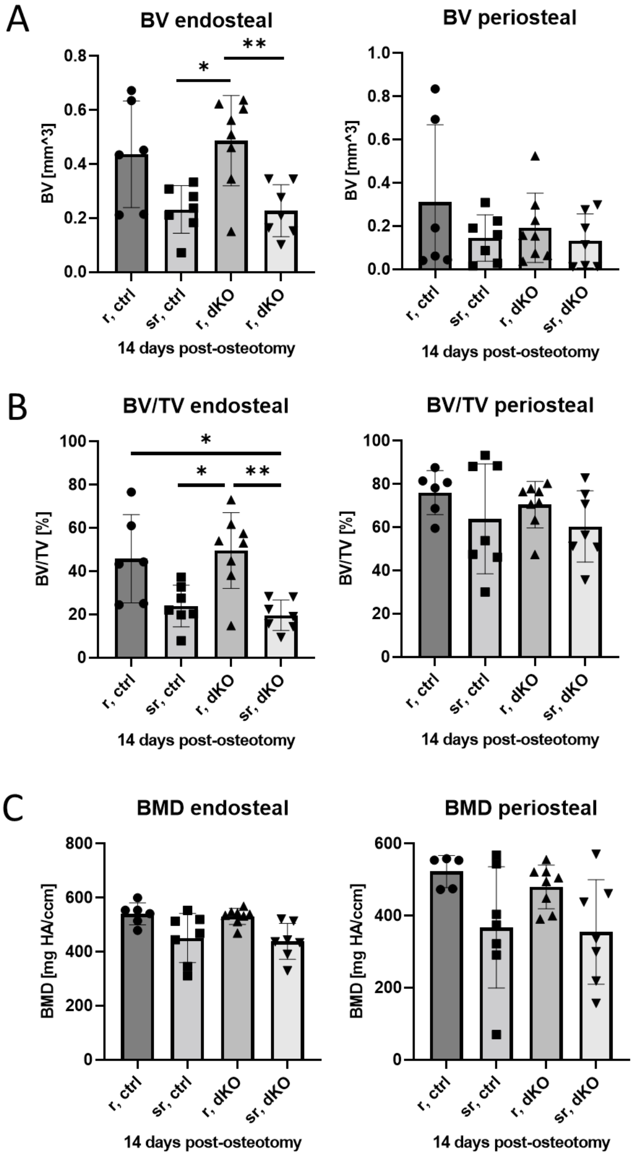


**Figure S6**. **Decreased mechanical stability led to reduced bone volume in the osteotomy gap in both, control and EC YAP/TAZ dKO mice.** µCT measurements were performed 14 days post-osteotomy for control and EC YAP/TAZ dKO under rigid vs semi-rigid fixation. Endosteal indicates the area within the osteotomy gap, whereas periosteal is the area outside next to the osteotomy gap. **A.** Within the osteotomy gap bone volume (BV) was significantly reduced under semi-rigid fixation compared to rigid. No difference could be detected between control and EC YAP/TAZ dKO mice. **B.** Concomitantly, bone volume of osteotomy gap over total volume of osteotomy gap was significantly reduced under semi-rigid fixation compared to rigid. **C.** EC YAP/TAZ dKO did not impact bone mineral density (BMD) nor did mechanical stability. Statistical analysis using one-way ANOVA followed by Tukey's test was performed. Different p values were indicated by * and ** for $p < 0.05$ and $p < 0.01$, respectively. (N=6-8)

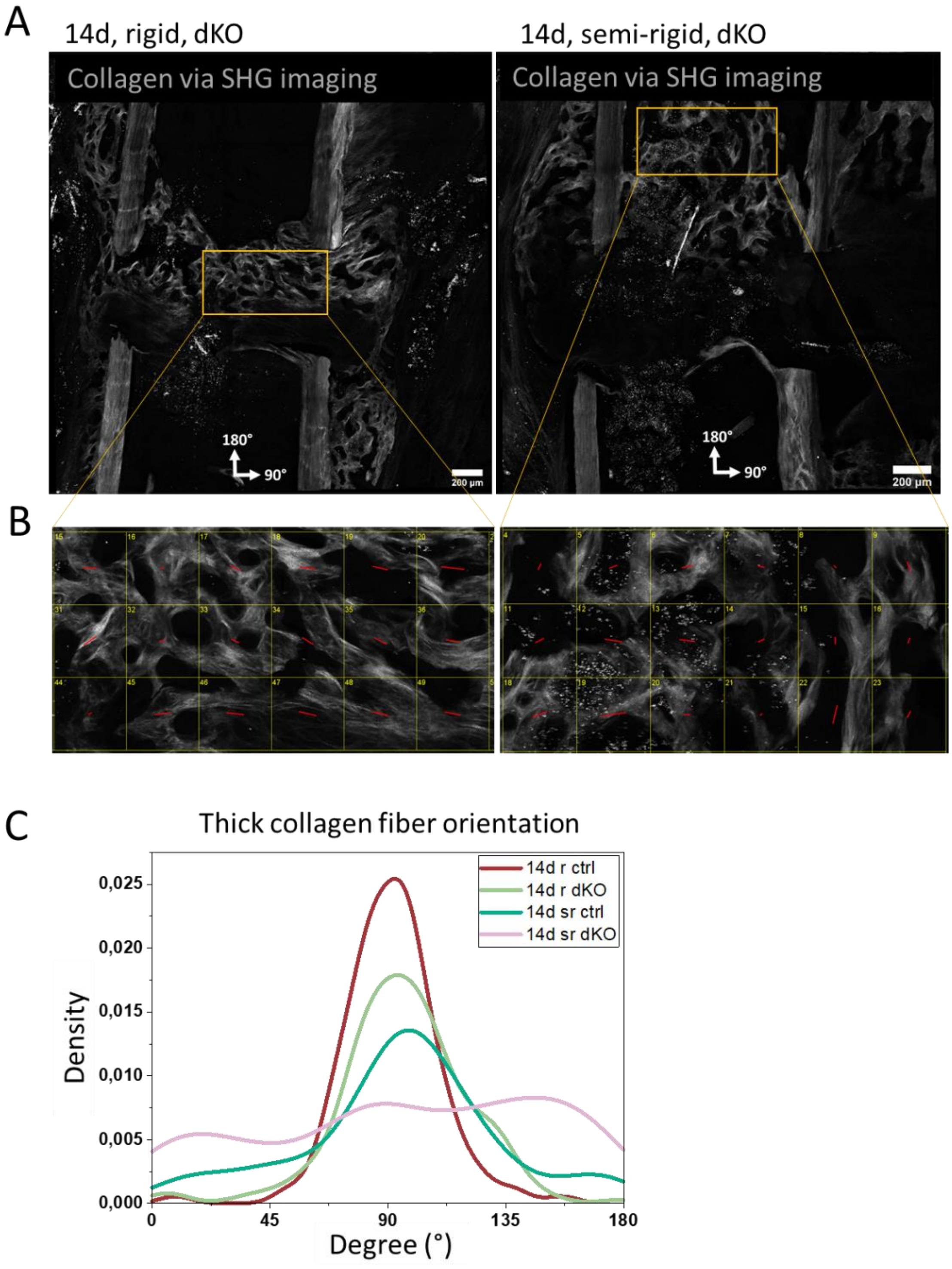


**Figure S7. Distribution of collagen fiber orientations in the osteotomy gap and in the proximal bone marrow cavity.** 180° is defined as parallel to the central bone axis and 90° degree perpendicular to the central bone axis. **A.** Representative SHG images for rigid and semi-rigid fixation 14 days post-osteotomy. Orientation analysis for collagen fibers within osteotomy gap and proximal bone marrow cavity were performed for all 14 days samples. **B.** Zoom-in images illustrate how our macro measured fibrillar collagen orientation. Collagen area was

divided into small region of interests. Average orientation of fibrillar collagen of each square was measured and depicted as a red line. The length of the line represents the degree of anisotropy. Average orientation of each square was then plotted into a graph. 180° is defined as the long bone axis. **C.** Diagram shows the distribution of collagen fiber orientation of all animals per group. Collagen fibers aligned more perpendicular to the central bone axis under rigid fixation compared to semi-rigid fixation in both, control and EC YAP/TAZ dKO mice (N=4-5 for rigid ctrl and dKO, N=6 for semi-rigid ctrl and dKO).

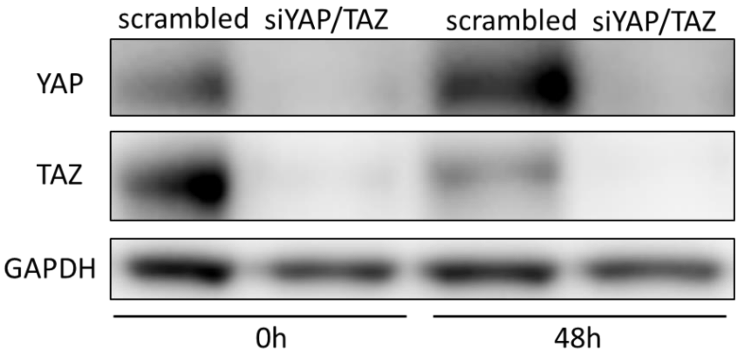


**Figure S8. Efficiency of YAP/TAZ knockdown in HUVECs**. Western blot of HUVECs performed at the start (0h) and at the end (48h) of collecting conditioned media. The start of collecting media was performed after 24h of siRNA transfection.

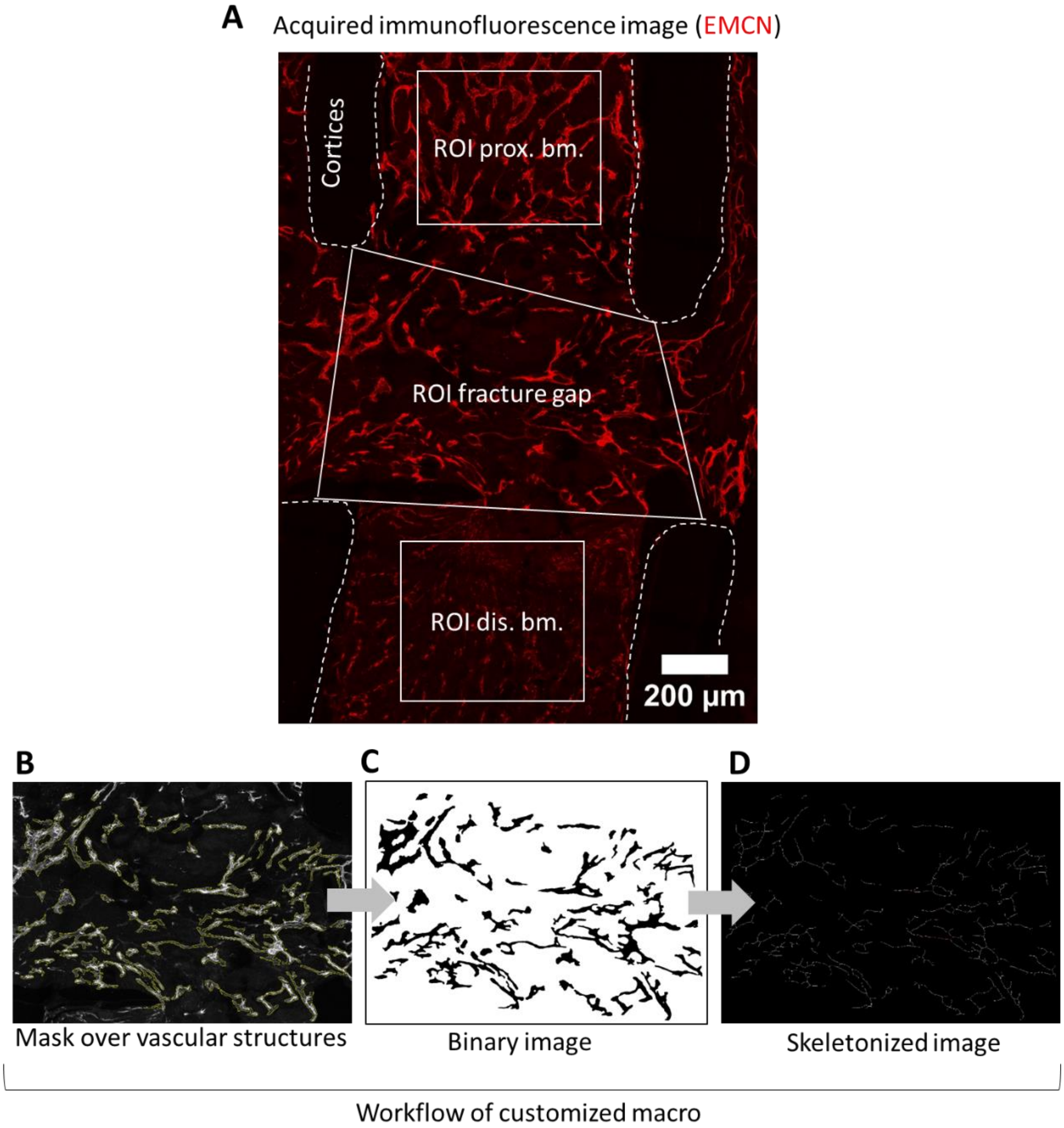


**Figure S9. Quantification of vessel density, vessel diameter and vessel orientations. A.** An illustration of the vessel quantification analysis is provided using an immunofluorescence image stained for EMCN to visualize vessels. Regions of interest (ROIs) were defined within the proximal and distal bone marrow. The ROI for the osteotomy gap was defined by connecting the midpoints of the four cortical fracture ends. **B-D.** Workflow of the custom macro used in FiJi is outlined along with an example of the osteotomy gap ROI. A mask was placed over the vascular structures **(B)** and then converted into a binary image **(C)**. Vessel density was calculated by measuring the total positive vessel area over the total osteotomy gap area from the binary image. Vessel diameter was measured for each vessel segment, and the mean vessel diameter was subsequently determined. A skeletonized image was generated, from which vessel orientation was calculated **(D)**.

| Gene | forward/reverse sequence |
| --- | --- |
| *HPRT1* | TATGGACAGGACTGAACGTC |
| | TGATGTAATCCAGCAGGTCA |
| *Col1a2* | AGCCGGAGATAGAGGACCAC |
| | GGCCAAGTCCAACTCCTTTT |
| *Col3a1* | GGGAGAAATGGTGACCCTGG |
| | TGCGAGTCCTCCTACTGCTA |
| *Col6a1* | ACTGCGCTATCAAGAAGGGG |
| | TCGTTCACAGCATCCTCCAG |
| *Lama5* | ACAGCAACAAGGCACACCC |
| | CAAACTTGATGAGGACGTAGGC |
| *Bmp1* | CTCCATCAAAGCTGCAGTTCC |
| | CGGGATCTACCTCTCCATCTC |
| *Timp1* | GCTTCTGGCATCCTGTTGTT |
| | ACGCTGGTATAAGGTGGTCTG |
| *Timp2* | CAAAGGGCCTGAGAAGGATA |
| | AGGCTCTTCTTCTGGGTGGT |
| *Mmp1* | ACATGAGTCTTTGCCGGAGG |
| | ATCCCTTGCCTATCCAGGGT |
| *Mmp8* | AACCAGCAACTACTCACTCCC |
| | GTGCTTGGTCCAGTAGGTTG |
| *Mmp13* | TTGAGCTGGACTCATTGTCG |
| | TCTCGGAGCCTCTCAGTCAT |

**Table S1. Primer sequences used for qPCR.**
